# A hypothalamic dopamine locus for psychostimulant-induced hyperlocomotion in mice

**DOI:** 10.1038/s41467-022-33584-3

**Published:** 2022-10-08

**Authors:** Solomiia Korchynska, Patrick Rebernik, Marko Pende, Laura Boi, Alán Alpár, Ramon Tasan, Klaus Becker, Kira Balueva, Saiedeh Saghafi, Peer Wulff, Tamas L. Horvath, Gilberto Fisone, Hans-Ulrich Dodt, Tomas Hökfelt, Tibor Harkany, Roman A. Romanov

**Affiliations:** 1 Department of Molecular Neurosciences, Center for Brain Research, Medical University of Vienna, Vienna, Austria; 2 Section for Bioelectronics, Center for Brain Research, Medical University of Vienna, Vienna, Austria; 3 Department of Bioelectronics, Institute of Solid State Electronics, Technical University of Vienna, Vienna, Austria; 4 Department of Neuroscience, Biomedicum, Karolinska Institutet, Solna, Sweden; 5 Department of Anatomy, Semmelweis University, Budapest, Hungary; 6 SE NAP Research Group of Experimental Neuroanatomy and Developmental Biology, Hungarian Academy of Sciences, Budapest, Hungary; 7 Department of Pharmacology, Medical University Innsbruck, Innsbruck, Austria; 8 Institute of Physiology, Christian-Albrechts-University, Kiel, Germany; 9 Department of Neuroscience, Department of Comparative Medicine, Yale School of Medicine, New Haven, CT USA

**Keywords:** Circadian rhythms and sleep, Molecular neuroscience, Motor control

## Abstract

The lateral septum (LS) has been implicated in the regulation of locomotion. Nevertheless, the neurons synchronizing LS activity with the brain’s clock in the suprachiasmatic nucleus (SCN) remain unknown. By interrogating the molecular, anatomical and physiological heterogeneity of dopamine neurons of the periventricular nucleus (PeVN; A14 catecholaminergic group), we find that *Th*^+^/*Dat1*^+^ cells from its anterior subdivision innervate the LS in mice. These dopamine neurons receive dense neuropeptidergic innervation from the SCN. Reciprocal viral tracing in combination with optogenetic stimulation ex vivo identified somatostatin-containing neurons in the LS as preferred synaptic targets of extrahypothalamic A14 efferents. In vivo chemogenetic manipulation of anterior A14 neurons impacted locomotion. Moreover, chemogenetic inhibition of dopamine output from the anterior PeVN normalized amphetamine-induced hyperlocomotion, particularly during sedentary periods. Cumulatively, our findings identify a hypothalamic locus for the diurnal control of locomotion and pinpoint a midbrain-independent cellular target of psychostimulants.

## Abstract (web summary)

The psychostimulant-sensitive neural mechanism linking the circadian clock to locomotion is unknown. Here, hypothalamic A14 neurons are shown to time diurnal activity by entraining the lateral septum, and their activity is shown to be sensitive to amphetamine.

## Introduction

The hypothalamus is an evolutionarily conserved brain region with its many distinct neural circuitries recognized for orchestrating peripheral endocrine signals in all vertebrates. The profound cellular heterogeneity of hypothalamic neuronal networks is seen as causal to their ability to orchestrate not only basic physiological needs, including sleep and wakefulness, reproduction, food or fluid intake^1–3^, but also complex behaviors, e.g., stress, aggression, sexuality, and parenting^4–11^. To this end, hypothalamic neuronal subtypes gain operational competence through combinations of fast neurotransmitters, neuropeptides, and hormones (including dopamine)^12–15^ whose permutations can generate functional specificity^16^.

Dopamine in the hypothalamus is best known for inhibiting prolactin release from the pituitary when released into the hypophyseal portal system in a diurnal fashion^17,18^. However, the presence of dopamine neurons within a patchwork of nuclei within the hypothalamus (A11, A12, A14, and A15 groups), as well as in adjacent hypothalamic/extrahypothalamic brain areas (e.g. A8–A11 and A13)^19–21^ suggests that molecularly segregated dopamine subtypes could differ in their intra- and/or extrahypothalamic circuit motifs and thus be critical to generate non-endocrine hypothalamic output. Such functional heterogeneity is faithfully illustrated by A12 neurons being a source of dopamine to inhibit prolactin release from the pituitary gland^5,18^ vs. A13 or A15 neurons. While the A13 locus regulates brown fat thermogenesis^3^, those in the anteroventral periventricular nucleus (A15 locus) control maternal care and oxytocin secretion^22^. Despite recent progress in recognizing the manifold roles of dopamine signaling, the existence of subtypes amongst A14 dopamine neurons, which are dispersed in the medial subdivision of the periventricular nucleus (PeVN) stretching along the third ventricle, and their differential contributions to either endocrine processes^13^ or too motivated behaviors via extrahypothalamic projections remain unexplored.

A14 neurons receive dense neuromedin S (NMS)^+^ innervation from the suprachiasmatic nucleus (SCN)^13^, suggesting a tentative position in the circadian pacemaker circuit^23,24^ and entrainment by a significant intrahypothalamic source^25–27^. However, if molecular diversity exists amongst A14 neurons that populate the PeVN affecting their synaptic efferent projections, cellular targets, and ultimately, function in any discrete neurocircuit is known. Here, we use virus-assisted circuit reconstruction in combination with intersectional mouse genetics and intact tissue microscopy to show that dopamine neurons in the anterior PeVN act as hypothalamic relays for the circadian clock circuit by projecting to the lateral septum (LS). This circuit layout is then implicated in the control of exploratory and locomotor activity, which undergo diurnal fluctuations^28–30^, and are phase-locked with the nocturnal nature of rodents. Lastly, we recognize that *Dat1* expression by dopamine neurons in the anterior PeVN could assign them as a cellular target for psychostimulants, particularly amphetamine, which are known to shift the circadian clock, thus inducing hyperlocomotion during time epochs naturally associated with resting (or even sleep)^31–36^. Indeed, recent brain-wide imaging suggests an amphetamine-sensitive locus in the periventricular hypothalamus^37^. We find that dopamine neuron in the PeVN account for the amphetamine-induced neuronal activation in the hypothalamus with their inactivation preventing amphetamine-induced hyperlocomotion during inactive periods when naïve rodent behavior is sedentary.

## Results

### Subclasses of A14 dopamine neurons

**Fig. 1:**
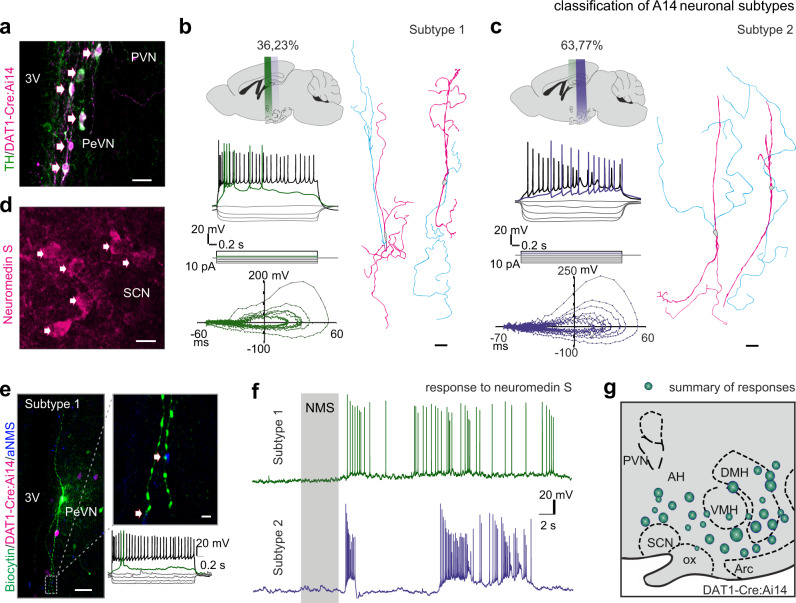
Morphological and functional classification of *Dat1*^+^ neurons in the PeVN. **a** Immunohistochemical identification of *Dat1*^+^ A14 dopamine neurons co-expressing tyrosine hydroxylase (TH) and tdTomato (fluorescence marked by *arrows*) in the PeVN of *Dat1*-Ires-Cre:Ai14 mice (*n* = 10 animals). Scale bar = 50 μm. **b** and **c** Representative current-clamp recordings of *Dat1*^+^ neurons in the PeVN and their post-hoc morphological reconstruction, including subtype “1” (**b**) and “2” (**c**) cells. *Top left*: schematic illustration showing the rostrocaudal allocation of A14 subtypes across the PeVN. The proportion of each neuronal subtype is indicated. *Middle left*: Action potential (AP) responses to two rheobase current pulses (*colored*) and at maximal frequency (*black*). *Bottom left*: Phase-plane plots of APs rising from 2× rheobase current injection. *Right*: Morphological reconstruction of a representative biocytin-filled *Dat1*^+^ neuron for each subtype. Axons were colorized in cyan, whereas dendrites were pseudocolored in magenta. **d** Immunohistochemistry for neuromedin S in the SCN (*n* = 9 animals). Scale bar = 10 μm. **e** Immunohistochemical detection of neuromedin S^+^ terminals contacting biocytin-filled *Dat1*^+^ neurons in the PeVN (*arrows*). Open rectangle denotes the location of the high-resolution inset. Representative AP signature identifies the target as a subtype “1” neuron. The experiment was performed in triplicate and produced invariable results. Scale bars = 50 μm (*left*) and 10 μm (*right*). **f** Representative current-clamp recordings of neuromedin-S induced AP firing by subtype “1” (*top*, *n* = 17 cells with 14 responders) and “2” neurons (*bottom*, *n* = 21 cells; 16 responders). Data were obtained in *n* = 14 animals. **g** Schematic map of A14 neuronal responses to neuromedin S in the rostrocaudal domain of the PeVN. Circle sizes are proportional to response strength (a total of *n* = 30 neurons from 14 animals were mapped). 3V third ventricle, AH anterior hypothalamus, Arc arcuate nucleus, DMH dorsomedial hypothalamus, PVN paraventricular nucleus, ScN suprachiasmatic nucleus, VMH ventromedial hypothalamus. Biorender was used to style experimental arrangements and brain regions (in **b**, **c**, **g**).

The molecular machinery for dopamine production relies on the enzymes tyrosine hydroxylase (TH) and aromatic-l-amino-acid decarboxylase (AADC), while its release requires vesicular monoamine transporter 2 (VMAT2) to load dopamine into synaptic vesicles^16,38^. The corresponding genes (*Th*, *Ddc*, *Slc18a2*) are considered phenotypic determinants of hypothalamic neuroendocrine neurons producing dopamine for systemic release at the median eminence (ME)^13,18,38–40^. In contrast, the plasmalemmal transporter for dopamine reuptake (DAT/*Dat1*) is less promiscuous in hypothalamic dopamine neurons^13^, and is only expressed by specific neuronal populations that do^5,13^ or do not produce dopamine^7^ (Supplementary Fig. 1a). Thus, we consider the coincident presence of *Th, Ddc, Slc18a2,* and *Dat1* as specific marks for A14 neurons in the PeVN that use dopamine for synaptic neurotransmission^13^ (Fig. 1a, Supplementary Fig. 1b, c). Here, we chose *Dat1*-Ires-Cre::Ai14 reporter mice combined with post-hoc TH histochemistry (Supplementary Fig. 1a) to correlatively test the morphological, physiological, and functional properties of A14 dopamine neurons, and specifically addressed if they could act as cellular interfaces of a circadian multi-synaptic loop that links the SCN to extrahypothalamic targets^13,41,42^.

Firstly, we performed patch-clamp recordings of TdTomato^+^ neurons spatially mapped along the longitudinal axis of the PeVN in acute brain slices (*n* = 17 mice, *n* = 54 cells). By combining electrophysiological analysis with the post-hoc three-dimensional reconstruction of biocytin-filled, TH-labeled TdTomato-positive(^+^) neurons, we identified two groups of intercalated *Dat1*^+^ A14 neurons, which we classified as subtype “1” and “2”, based on their action potential (AP) waveforms and frequency, location, and extent of dendritic arborization (Fig. 1b, c, Supplementary Fig. 2a). Rostro-caudally, subtype “1” neurons are present at the level of the SCN. In contrast, subtype “2” neurons are located more posterior, with their accumulation peaking at the level of the arcuate nucleus. Electrophysiologically, subtype “1” neurons are distinguished by the ability to generate initial bursts upon current injection (2.68 ± 0.24 AP/burst, *n* = 22; Fig. 1b), whereas subtype “2” cell are characterized by being tonically depolarized upon step current injection, the accommodation of APs (Fig. 1c), and spontaneous bursting (Supplementary Fig. 2b). As such, these electrophysiological properties resemble those of classical tuberoinfundibular dopamine (TIDA) neurons^43,44^.

### A14 dopamine neurons are ubiquitously innervated by the SCN

Earlier single-cell RNA-seq studies showed that dopamine neurons in the PeVN express receptors for neuromedins (*Nmbr, Nmur2*), nociceptin, and PACAP^13^. Among these, abundant *Nmur2* expression (100% of cells with 3.17 ± 1.25 RNA molecules/cell^13^, Supplementary Fig. 1c) suggests sensitivity to its cognate ligand, NMS. NMS is produced in a circadian fashion^13,42^ within the SCN (Fig. 1d). Here, we show that NMS^+^ terminals indeed innervate dendrites of most A14 neurons reconstructed after post-hoc identification, including both subtype “1” and “2” cells (Fig. 1e, Supplementary Fig. 2c). These data define SCN-to-PeVN innervation targets, which hitherto were ambiguous^13^.

Next, and considering the electrophysiological and morphological heterogeneity of dopamine neurons (Fig. 1b, c), we asked whether diurnal differences in NMS responsiveness could exist between the two subtypes. Our electrophysiological experiments revealed that 80% of neurons from both electrophysiological subtypes (15/18 cells (subtype “1”) and 20/25 cells (subtype “2”)) were activated by exogenous NMS superfusion (Fig. 1f, g, data were summarized for recordings performed during the day and at night) but not by superfusion of control bath solution (Supplementary Fig. 2d). Next, we confirmed that NMS levels indeed vary in the SCN^13^ in a circadian manner, with a significant increase (Supplementary Fig. 1e, f) during late-night hours^42^. Accordingly, the spontaneous activity of A14 dopamine neurons in acute vibroslices increased during dark hours (circadian time (CT): 18:00–23:00) for both subtypes (Supplementary Fig. 2g). Altogether, we suggest that despite the internal heterogeneity of dopamine cells in the PeVN, they all are directly entrained by SCN afferents^27^, respond to NMS, and are diurnally regulated at least ex vivo. Overall, at the level of SCN input specificity, the two subtypes of A14 dopamine neurons do not differ. Nevertheless, their segregation along the rostrocaudal axis of the PeVN could confer differences in axonal targeting, that is projections to different anatomical locations in- and outside the hypothalamus. If so, one might also posit that extrahypothalamic outputs of A14 neuron subtypes could underpin diurnal variations in higher brain functions.

### Brain-wide mapping of dopamine efferents identifies the LS as a target for subtype “1” neurons

**Fig. 2:**
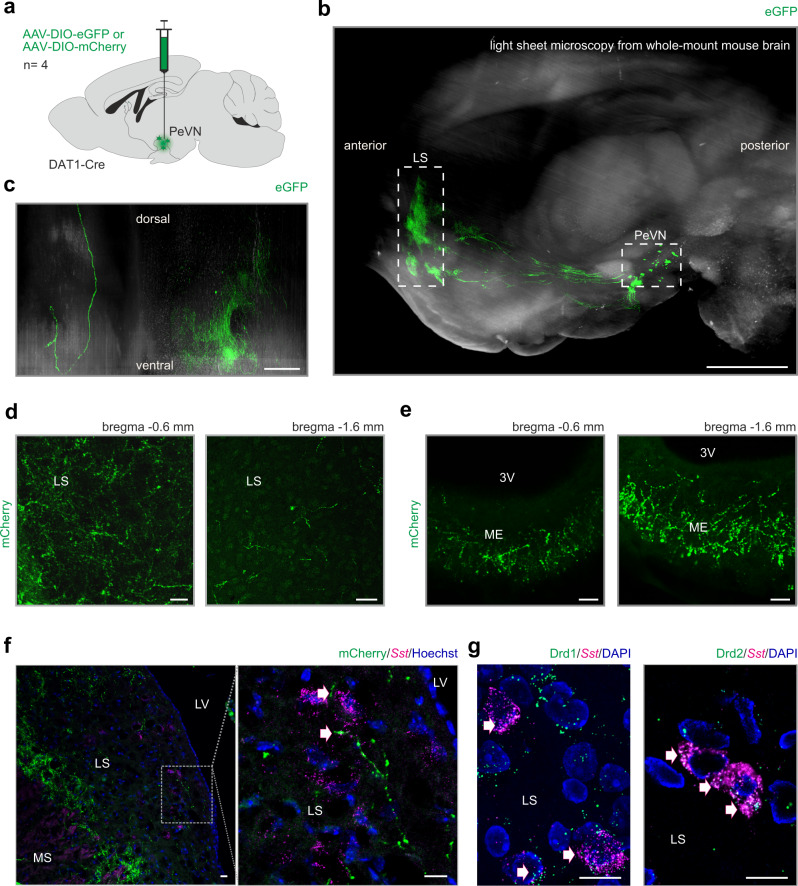
Extrahypothalamic targets of *Dat1*^+^ neurons populating the PeVN. **a** Graphical rendering of the experimental design for anterograde viral labeling of the projections of *Dat1*^+^ neurons by using AAV-hSyn-DIO-mCherry and AAV-hSyn-DIO-EGFP.WPRE.hGH shown in (**b–f**). **b** Sagittal brain view to depict efferent fibers originating from A14 neurons as revealed by light-sheet microscopy. Dashed rectangle identifies the lateral septum (LS), a main extrahypothalamic target of A14 neurons (*n* = 4 animals). Scale bar = 1 mm. **c** High-resolution image of A14 projections towards the LS. Scale bar = 0.2 mm. **d** and **e** Axonal projections of subtype “1” (anterior) and “2” (posterior) DAT^+^ neurons to the LS (**d**) and median eminence (**e**), respectively. Scale bars = 20 μm. **f** In situ hybridization for *Sst* in the LS, combined with the immunohistochemical localization of neuronal projections from virus-infected *Dat1*^+^ neurons of the PeVN (*n* = 4 animals). Scale bar = 10 μm. **g** In situ hybridization for *Sst* co-localized with both *Drd1-GFP* and *Drd2-GFP* in septal neurons. Scale bar = 10 μm. 3V third ventricle, LS lateral septum, LV lateral ventricle, ME median eminence. We used Biorender to draw the experimental scheme in (**a**).

The circuit function of A14 dopamine neurons is unclear. Previous data show that some A14 dopamine neurons^13^, alike other secretory command neurons (e.g. A12)^5^, project to the ME and participate in dopamine secretion into the hypophyseal-blood portal system to control prolactin release from the hypophysis^18,19^. Yet, brain-wide mapping of A14 output has yet to be performed to define if subtype-specific target segregation exists. To this end, we stereotaxically injected Cre-dependent GFP-producing AAV2 viral particles into the PeVN of *Dat1*-IRES-Cre mice (Fig. 2a) and used tissue clearing and ultramicroscopy (light-sheet microscopy) to map individual axons at distant locations^45,46^. We found that A14 neurons give rise to long projections that leave the hypothalamus and densely innervate the LS (Fig. 2b, c, Supplementary Fig. 3a, Supplementary Video 1, *n* = 4 animals), with additional efferents coursing towards the central amygdaloid nucleus and the ME (Supplementary Video 2). To distinguish the participation of subtype “1” and “2” A14 dopamine neurons in establishing zonal innervation, we compared the distribution of axonal projections by spatially segregated tracing of either subtype “1” (40 nl virus injections at the SCN level, bregma = −0.6 mm; see Supplementary Fig. 3b, c) or subtype “2” cells (40 nl virus injections at the level of the periventricular nucleus, bregma = −1.6) in *Dat1*-Ires-Cre mice. The experimental approach allowed us to exclusively map subtype “1” neurons (Supplementary Fig. 3b, c) and show that these cells innervate the LS (Fig. 2d). In contrast, subtype “2” neurons predominantly projected to the ME (Fig. 2e) suggesting their primary involvement in regulating prolactin secretion. In the rest of this study, we exclusively focused on the LS-projecting subset of subtype “1” neurons, because earlier results suggest the involvement of the LS in the control of motor activity in rodents^29,47,48^. However, to the best of our knowledge, earlier data did not identify either the cellular origins of a clock network that would provide upstream regulation to LS neurons or the specific neuronal contingent within the LS that is directly entrained by hypothalamic efferents.

Based on previous tract-tracing and functional studies suggesting that somatostatin (*Sst*)^+^ neurons of the LS receive particularly rich synaptic inputs from non-identified non-tegmental dopamine neurons^30,49,50^, we hypothesized that dopamine neurons of the anterior PeVN could form any such projection. Here, we combined in situ hybridization to visualize *Sst*^+^ neurons in the LS (*n* = 4 animals) and anti-mCherry immunohistochemistry to localize nerve endings of A14 dopamine neurons that were virally transduced to express mCherry in a *Cre* recombinase-dependent fashion (Fig. 2a, f and Supplementary Fig. 3d). These experiments revealed mCherry^+^ nerve endings in close apposition to 48.21 ± 2.47% of *Sst*^+^ LS neurons (Fig. 2f, but see also high-resolution laser scanning microscopy for SST; Supplementary Fig. 3d), which themselves are long-axon principal GABA cells receiving inputs from the hippocampus and innervating the ventral tegmental area and ventral forebrain (nucleus accumbens, olfactory tubercle, and the substantia innominata)^47,50,51^.

For *Sst*^+^ neurons to decode dopamine signals, they ought to express either D1 or D2 dopamine receptors, or both. Here, we used D1-EGFP and D2-EGFP reporter mice^52,53^ to show that *Sst*^+^ neurons are indeed GFP^+^, suggesting their ability to respond to dopamine. To reinforce this notion, we show that virally traced A14 projections indeed appose *Sst*^+^ neurons co-expressing mRNAs for *Drd1* and *Drd2* (Fig. 2f, g, Supplementary Fig. 3d, e). These data support the existence of an extrahypothalamic dopaminergic pathway terminating on *Sst*^+^ GABA neurons of the LS.

**Fig. 3:**
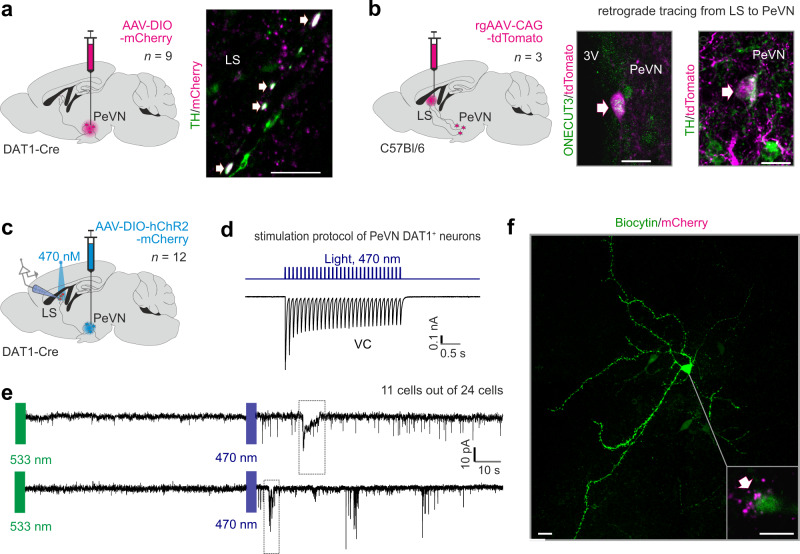
Subtype “1” *Dat1*^+^ neurons in the anterior PeVN innervate the lateral septum. **a** Tyrosine hydroxylase (TH) is present in neuronal projections coursing towards the lateral septum (LS). *Left*: experimental scheme. *Right:* immunohistochemistry for TH and mCherry in fibers projecting from the PeVN to the LS. Immunohistochemistry was performed for each viral injection (*n* = 9 independent experiments) with similar results. Scale bar = 10 μm. **b** TH^+^/Onecut3^+^ neurons situated in the PeVN were labeled retrogradely (*left*) by stereotaxic injections targeting the LS. Onecut3 localization is shown to the *right*. Immunohistochemistry was performed for each viral injection (*n* = 3 independent experiments) and yielded similar results. Scale bars = 20 μm. **c** Experimental scheme for ex vivo electrophysiological circuit mapping **e** with optogenetic stimulation (**d**; *n* = 12 animals). **d** Periventricular *Dat1*^+^ neurons infected with channelrhodopsin-2 (ChR2) generate inward currents upon repetitive light stimulation. **e** Patch-clamp recordings of neurons in the LS show their activation (11 out of 24 cells) upon light stimulation of ChR2-expressing *Dat1*^+^ terminals. **f** Post-hoc reconstruction of a biocytin-filled neuron responding to optogenetic stimulation **e** confirmed synaptic innervation by *Dat1*-Cre^+^ (mCherry-filled) afferents originating from the PeVN. Scale bar = 50 μm. 3V third ventricle. Biorender assisted us to draw schemes and anatomical structures (in **a**–**c**).

To substantiate that feed-forward dopaminergic neurotransmission exists between A14 neurons and their *Sst*^+^ targets in the LS, we examined if nerve terminals from A14 dopamine neurons of *Dat1*-Ires-Cre mice that had been stereotaxically transduced to express AAV-DIO-mCherry (bregma = −0.6 mm) contain TH and/or VMAT2 (*Slc18a2*). Indeed, we revealed the presynaptic co-localization of both TH (Fig. 3a) and VMAT2 (Supplementary Fig. 4a), coincidently required for the synthesis and vesicular release of dopamine, with mCherry in the LS.

Lastly, we performed retrograde tracing by injecting AAV-CAG-tdTomato viral particles into the LS (Fig. 3b, Supplementary Fig. 4b). We identified tdTomato^+^ cell bodies in the anterior PeVN, which were co-labeled with either TH or *Onecut3* (Fig. 3b), the latter being a transcription factor selectively expressed by of subtype “1” dopamine neurons in the PeVN^40^. Together, our reciprocal Cre-dependent axonal mapping and brain-wide imaging data highlight that *Dat1*^+^/*Onecut3*^+^ dopamine neurons in the anterior PeVN project to the LS and harbor the enzymatic and release machinery to use dopamine as neurotransmitter in a projection pathway targeting *Drd1*^+^/*Drd2*^+^/*Sst*^+^ GABA neurons in the LS.

### Dopamine efferents from the PeVN modulate neuronal activity in the LS

To demonstrate that a PeVN-to-LS projection operates with dopamine as neurotransmitter acting at metabotropic GPCRs, we injected Cre-dependent AAV viruses co-expressing channelrhodopsin-2 (hChR2) and mCherry (AAV-DIO-hChR2-mCherry) into the PeVN and used a combination of slice electrophysiology and in vitro optogenetics for circuit mapping (Fig. 3c–e, Supplementary Fig. 4c–f; for direct activation of dopamine neurons in PeVN see Fig. 3d). Simultaneous light stimulation of the LS and probing of individual neurons by patch-clamp electrophysiology (*n* = 24) showed that optogenetic activation of nerve terminals originating from the anterior PeVN evoked transient inward currents (max amplitude −43.88 ± 6.69 pA for blue (470 nm) but not green (533 nm) light stimulation vs. −9.53 ± 1.71 pA in control) and increased the frequency of spontaneous currents (3.44 ± 0.71 Hz vs. 0.56 ± 0.1 Hz in control) in 11/24 neurons (Fig. 3e). Control recordings with blue light had no effect on either the induction or frequency of currents (Supplementary Fig. 3f). The fact that optogenetic stimulation remained efficient even in the presence of tetrodotoxin (Supplementary Fig. 4c) reinforced the monosynaptic action of dopamine released onto the recorded cells^54^. Anatomical support for the above conclusions was obtained by post-hoc immunohistochemistry showing that ChR2^+^ nerve endings contacted LS neurons that we had probed electrophysiologically (Fig. 3f). The optogenetic activation that persisted in the presence of tetrodotoxin (Fig. 3e, Supplementary Fig. 4c) was relatively slow, which suggests synaptic signaling through metabotropic rather than fast ionotropic receptors^55–57^. This concept is compatible with the molecular and morphological signature of dopamine neurons establishing both targeted innervation and en passant synapses in the LS, allowing for the broad-range volumetric activation of D1 and D2 G-protein-coupled receptors^58,59^. Likewise, our observations support a primarily metabotropic mechanism, unlike neurotransmitters activating ionotropic receptors at the millisecond range^55^.

### Chemogenetic dissection of dopamine neurotransmission in the LS

**Fig. 4:**
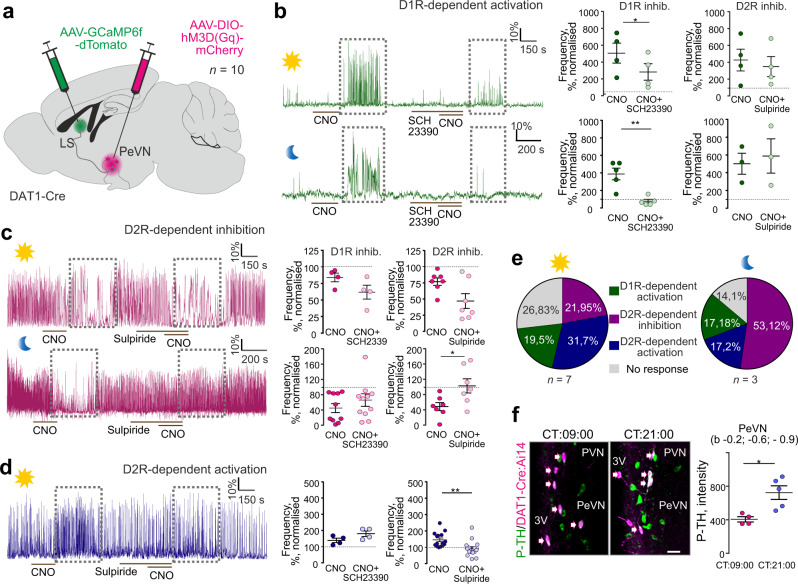
Subtype “1” *Dat1*^+^ neurons in the anterior PeVN modulate neuronal activity in the LS. **a** Experimental design of virus injections for the ex vivo pharmacogenetic mapping of the PeVN-LS circuitry. **b–d** CNO stimulation of *Dat1*^+^ terminals innervating the LS alters local Ca^2+^ oscillations. Colored traces (*left*) show representative examples of Ca^2+^ recordings at the single-cell level and their sensitivities to dopamine receptor antagonists. Day (sun symbol) and night (moon symbol) correspond to the timing of particular experiments (CT: 06:00–11:00 and 18:00–23:00, respectively). *Right*: Effect of D1 and D2 dopamine receptor antagonism by SCH23390 and sulpiride, respectively, on CNO-induced Ca^2+^ oscillations. Data were expressed as means ± s.e.m., with individual values also shown. Two-sided paired *t*-test was used for statistical analysis; *p < 0.05, **p < 0.01; *n* indicates the number of cells recorded from for each response type over 7 animals during the day and 3 animals during the night. In **b** at daytime: D1R inhibition: *n* = 4 cells, *p* = 0.0101; D2R inhibition: *n* = 4 cells, *p* = 0.1282; at nighttime: D1R inhibition: *n* = 5 cells, *p* = 0.0062, D2R inhibition: *n* = 3 cells, *p* = 0.668. In **c** for day recordings: D1R inhibition: *n* = 4 cells, *p* = 0.141; D2R inhibition: *n* = 7 cells, *p* = 0.0205; for night recordings: D1R inhibition: *n* = 11 cells, *p* = 0.915; D2R inhibition: *n* = 7 cells, *p* = 0.0306. In **d** during daytime: D1R inhibition: *n* = 4 cells, *p* = 0.1275; D2R inhibition: *n* = 15 cells, *p* = 0.0041. **e** Four neuronal populations were shown to exist in the LS, each responding differently to the chemogenetic activation of *Dat1*^+^ inputs from the PeVN. The distribution of functional groups differs during the day vs. night phases with an increased fraction of non-responsive neurons detected during the day. *n* states the number of independent experiments. **f** Levels of phospho-Ser^40^-TH at day and night. Scale bar = 20 μm. Data were statistically evaluated by using two-sided unpaired *t*-test and presented as means ± s.e.m. *p* = 0.0119 (*p < 0.05); *n* = 4 animals for CT 09:00 and 5 animals for CT 21:00. 3V third ventricle, PeVN periventricular nucleus, PVN paraventricular nucleus. We used Biorender to visualize an experimental scheme.

Next, we assessed the impact of chemogenetically manipulating A14 dopamine neurons on the activity of LS. For this, we first infected the anterior PeVN of *Dat1*-Ires-Cre mice with a virus encoding AAV-DIO-hM3D(G_q_)-mCherry to enrich excitatory DREADDs in synaptic terminals in the LS^60^. Three days later, we infected LS neurons with a Cre-independent virus locally to read out Ca^2+^ signals by gCAMP6f, a genetically encoded Ca^2+^ indicator^61^ whose synapsin-1 promoter ascertained neuronal enrichment (Fig. 4a). This experimental design allowed us to specifically activate A14 projections ex vivo in slices containing the LS, with neuronal activity therein monitored by Ca^2+^-imaging (as green fluorescence). Considering that A14 dopamine neurons are entrained by the core circadian pacemaker, we hypothesized that their activity could change diurnally^13,42^. Thus, we performed two identical sets of experiments during the light (CT: 06:00–11:00) and dark phases (CT: 18:00–23:00). Bath-applied clozapine-N-oxide (CNO) altered neuronal activity, producing either activation or inhibition, in 70–85% of LS neurons during both time periods (Supplementary Fig. 5a–c). CNO remained ineffective in control recordings performed on brain slices from sham-injected animals (Supplementary Fig. 5d).

We then used a pharmacological approach to dissect the D_1_/D_2_ receptor selectivity of hypothalamic dopamine drive in the LS. This led us to distinguish three target neuronal cohorts in the LS (Fig. 4b–d, Supplementary Fig. 5a–c): (i) silent neurons activated upon CNO application (day: *n* = 16, oscillation frequency increased to 455.8 ± 53.14%, *p* < 0.001, amplitude: 300.3 ± 68.37%, *p* < 0.05; night: *n* = 11, oscillation frequency increased to 403.4 ± 47.15%, *p* < 0.001, amplitude: 197.9 ± 46.91%, *p* < 0.05; Supplementary Fig. 5a). These effects were blocked by SCH23390 but not sulpiride, D_1_ and D_2_ receptor antagonists, respectively (Fig. 4b). Since D_1_ receptors are excitatory^62^, we suggest direct dopamine action on these postsynaptic neurons. (ii) Neurons whose activity was significantly inhibited by CNO (day: *n* = 18, oscillation frequency decreased to 73.98 ± 2.7%, *p* < 0.001, amplitude: 100 ± 6.26%, *p* = 0.99; night: *n* = 33, oscillation frequency decreased to 58.66 ± 4.56%, *p* < 0.001, amplitude: 106.2 ± 8.25%, *p* = 0.45; Supplementary Fig. 5b). This action was blocked by sulpiride (at D_2_ receptors; Fig. 4c) but only during night. This finding suggests that alternative neurotransmitters might be recruited during the daytime. (iii) Neurons with increased activity in a D_2_ receptor-dependent manner (day: *n* = 26, oscillation frequency increased to 149.8 ± 8.01%, *p* < 0.001, amplitude: 93.65 ± 3.85%, *p* = 0.12; night: *n* = 11, oscillation frequency increased to 181.7 ± 23.86%, *p* < 0.01, amplitude: 128.5 ± 9.23%, *p* < 0.05; Fig. 4d and Supplementary Fig. 5c, d). Since D_2_ receptors are inhibitory in neurons^62^, we propose the indirect activation of these cells by recruitment of inhibitory neurons (feed-forward inhibition).

Likewise, we found that the allocation of neurons to the specific groups differed during day and night, with an increasing fraction of non-responsive cells during daytime (corresponding to inactive epochs; Fig. 4e, Supplementary Fig. 5f). This finding provides direct evidence for the ability of A14 neurons to undergo cell state-changes in response to a physiological stimulus. Accordingly, phospho^40^-TH levels significantly increased during night hours (Fig. 4f, Supplementary Fig. 5g), a histochemical surrogate of TH enzymatic activity^63,64^. This observation also correlates with NMS dynamics (Supplementary Fig. 2e, f), the diurnal activity (Supplementary Fig. 2g), and night-only effects of sulpiride-mediated inhibition of A14 dopamine neurons (Fig. 4c), suggesting a shift between day and night modes for A14 neurons through the tuning of dopamine neurotransmission.

### GABA neurotransmission is dispensable for LS entrainment

Previously, we showed that hypothalamic dopamine cells originate from GABAergic precursors and retain the expressional machinery to produce and release GABA even in adulthood^13,40^. Indeed, most *Th*^+^/*Onecut3*^+^ dopamine neurons in the PeVN harbor *Gad1* (70%), *Gad2* (88%), and *Slc32a1* (88%) postnatally (Supplementary Fig. 6a). At the same time, TH/VGAT double-positive synapses exist in the LS (Supplementary Fig. 6b), prompting the question if the coexistence of dopamine and GABA in the same terminals in the LS reflects the neurochemical heterogeneity of A14 dopamine efferents^39,56,65^.

When selectively assessing dual-neurotransmitter production at the protein level, VGAT localization within virally traced projections of dopamine neurons situated in the anterior PeVN showed only sporadic co-localization of VGAT with mCherry (2.9 ± 1%; Supplementary Fig. 6c). This finding suggests a remarkably minor contribution of GABA neurotransmission in PeVN-to-LS communication during either day or night. Accordingly, bicuculline, a GABA_A_ receptor antagonist, did not affect the CNO-induced activation of excitatory neurotransmission from *Dat1*^+^ nerve terminals in the LS ex vivo (Supplementary Fig. 6d). These data reinforce the primary role of dopamine in entraining LS neuronal ensembles.

### Dopamine neurons in the anterior PeVN modulate spontaneous locomotion

AP firing of neurons in the LS correlates with speed and acceleration during locomotion^28^. In contrast, electrolytic lesions of the LS produce hyper-reactivity in rodents^29,48,66^. More specifically, recent studies show that *Sst*^+^ interneurons of the dorsal LS regulate animal mobility^47^. Therefore, we hypothesized that A14 dopamine neurons could be central to the diurnal regulation of locomotor epochs.

**Fig. 5:**
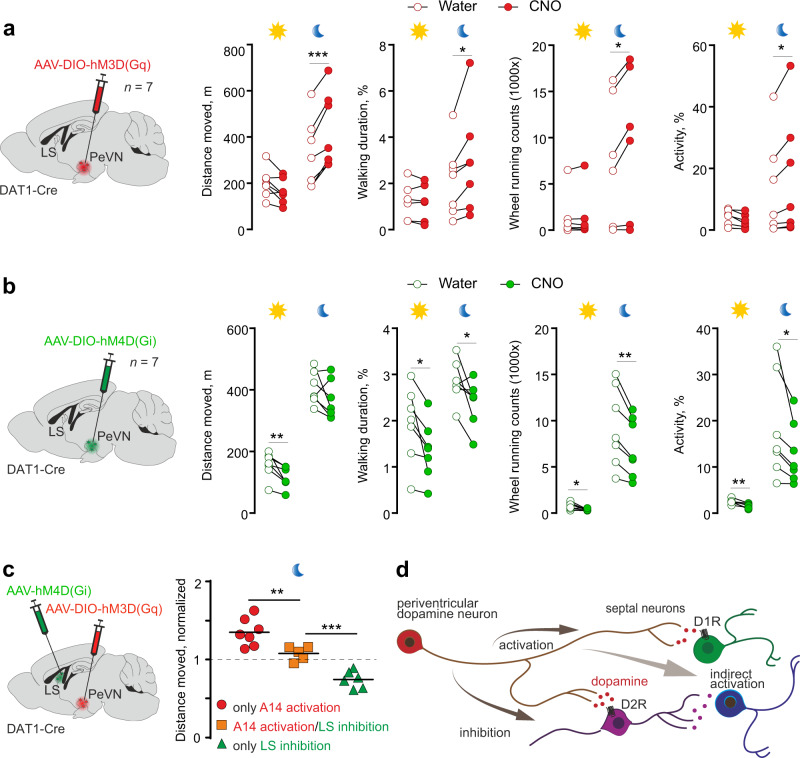
*Dat1*^+^ neurons residing in the PeVN modulate locomotion. **a** Chemogenetic stimulation of periventricular *Dat1*^+^ neurons led to an increase in locomotor activity during the dark phase (*n* = 7 animals, CT: 12:00-18:00) when infected with and stimulated by an AAV encoding hM3D(G_q_) and subsequent exposure to CNO. One-sided paired *t*-test was used to assess statistical significance in response to CNO administration. *P* values at night are 0.0001, 0.016, 0.012, 0.019 for “Distance moved, “Walking duration”, “Wheel running counts” and “Activity”, respectively. **b** Chemogenetic inhibition of periventricular *Dat1*^+^ neurons significant reduced locomotion during both the dark (CT: 12:00–18:00) and light (CT: 00:00–06:00) phases (*n* = 7 mice). *Dat1*^+^ neurons in the PeVN were infected with an AAV encoding hM4D(G_i_) and subsequently stimulated with CNO. One-sided paired *t*-test was used to assess statistical differences in CNO administration. *P* values are 0.0049, 0.0136, 0.0128, 0.0010 during the day for “Distance moved, “Walking duration”, “Wheel running” and “Activity”, respectively. For the dark period, respective *p* values for “Walking duration”, “Wheel running counts” and “Activity” are 0.0285, 0.0016, 0.0201. **c** Combinatorial chemogenetic experiments involving the simultaneous metabotropic activation of *Dat1*^+^ neurons in the PeVN and inhibition of LS neurons significantly reduced locomotion (orange rectangles, *n* = 5) as compared to when only activating *Dat1*^+^ neurons (red circles) to compensate for the inhibitory effect of the reduced activity of LS neurons (green triangles, *n* = 6). during the dark phase (CT: 12:00–18:00). One-way ANOVA with the Student–Newman–Keuls method for pairwise multiple comparisons was used returning *p* values as 0.004 (**), 0.001 (***). **d** Schematic illustration of functionally diverse LS neuron pools and their connectivity based on their differential pharmacological responses in Ca^2+^ imaging experiments. ***p < 0.001; **p < 0.01, *p < 0.05. We used Biorender for the design of experimental schemes and anatomical drawings (in **a**–**c**).

To test this hypothesis, we either chemogenetically activated (Fig. 5a, Supplementary Fig. 7a, b) or inhibited (Fig. 5b) dopamine neurons in the anterior PeVN and assessed spontaneous locomotor activity (that is, mobility in the home cage including in a running wheel but without external stressors) with emphasis on (i) walking duration, (ii) distance moved, (iii) frequency of wheel running, and (iv) the percentage of activity upon continuous monitoring of baseline and CNO-induced activities (CNO was made available in drinking water) each registered for 3 days. Locomotor activity was separately analyzed during both light and dark phases.

We found that the conditional activation of dopamine neurons significantly increased locomotion, particularly during the dark phase (Fig. 5a, *p* < 0.05 for all recorded parameters). In turn, DREADD-induced inhibition of dopamine neurons in the anterior PeVN suppressed locomotion (Fig. 5b, *p* < 0.05 for all recorded parameters except distance moved). In control experiments (Supplementary Fig. 7c, d), CNO alone in naïve animals or in Cre-expressing mice without DREADD vectors failed to impact locomotion. These findings support the contribution of dopamine A14 neurons to the diurnal control of locomotion as circadian effectors upstream of the LS (Fig. 5a, b).

Next, we tested the hierarchical relationship of the PeVN and LS neurons in the control of locomotion. To do so, we have tested locomotion after chemogenetically manipulating LS neurons through the focal delivery of either Cre-independent AAV-hM4D(Gi) or AAV-hM3D(Gq) (Supplementary Fig. 7e–g). We found that chemogenetic inhibition of LS neurons reduced locomotion during the active phase (CT: 00:00–06:00). Chemogenetic activation of LS neurons did not alter locomotion (Supplementary Fig. 7g), which we argued as a likely output given the many counteractions of septal neurons. Subsequently, we combined the Cre-dependent activation of dopamine neurons in the anterior PeVN and simultaneous chemogenetic inhibition of LS neurons (Fig. 5c). CNO exposure significantly reduced locomotion upon combined viral transduction, relative to its typical increase upon chemogenetically activating dopamine neurons in the PeVN alone (Fig. 5c, Supplementary Fig. 7g). These data cumulatively suggest that the LS is subordinate to the PeVN in the neurocircuit controlling locomotion.

### Amphetamine-induced activation of A14 dopamine neurons triggers hyperlocomotion

**Fig. 6:**
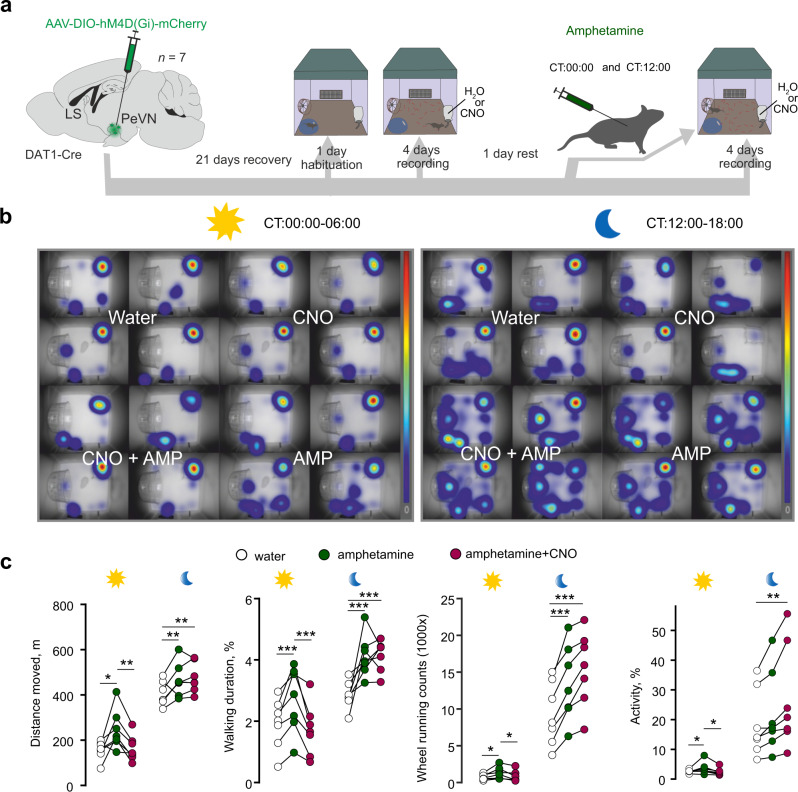
The contribution of *Dat1*^+^ neurons of the anterior PeVN to driving amphetamine-induced hyperlocomotion. **a** Experimental design that combines amphetamine administration with chemogenetic inhibition of *Dat1*^+^ neurons in the PeVN. **b** Experimental data on locomotor activity (heat map of animal positions in the home cage; distance moved; active time) upon amphetamine administration with/without the chemogenetic inhibition of *Dat1*^+^ neurons in PeVN. All results were separated for the day (light phase; CT 00:00–06:00) and night (dark phase; CT: 12:00–18:00). **c** Amphetamine significantly increased locomotor activity. The coincident chemogenetic inhibition (hM4D(G_i_)) of *Dat1*^+^ neurons in the PeVN occluded amphetamine-induced hyperlocomotion during the light (passive) phase (*n* = 7 mice/group). A one-way repeated-measures ANOVA was used to statistically analyze results with the Student–Newman–Keuls post-hoc method for pairwise multiple comparisons. ***p < 0.001; **p < 0.01, *p < 0.05. Statistically, significant *p* values were listed from left to right. For distance moved, daytime: *p* = 0.011(*), 0.007 (**); nighttime: *p* = 0.006 (**) and 0.006 (**). For walking duration, daytime: *p* = 0.001(***), <0.001 (***); nighttime: *p* = 0.001 (***) and 0.001 (***). For wheel running, daytime: *p* = 0.02(*), 0.019 (*); nighttime: *p* < 0.001 (***) and 0.001 (***). For activity, daytime: *p* = 0.039 (*), 0.045 (*); nighttime: 0.002 (**). We used Biorender to visualize an experimental scheme.

The inhibition of DAT is considered a key mechanism for psychostimulants, e.g., methylphenidate or amphetamine, to affect locomotion^67–69^, and to alter day vs. night activity^35,36,67^. Given those A14 dopamine neurons in the anterior PeVN express *Dat1*, these neurons could be recruited by psychostimulants to produce sustained excitation of the LS and thus increase motility. This mechanism could particularly be overridden by psychostimulants during the day when nocturnal rodents are generally immobile, and the network is at its ground state. We tested this hypothesis in mice that received intraperitoneal injections of amphetamine twice daily (CT: 00:00/12:00) with or without chemogenetic inhibition of dopamine neurons populating the anterior PeVN (Fig. 6a). Amphetamine significantly increased locomotor activity in control mice during both day (passive) and night (active) phases, recapitulating earlier findings^67,70,71^. Notably, chemogenetic inhibition of A14 dopamine neurons during the lit but not dark phase (Fig. 6b, c) significantly attenuated amphetamine-induced hyperlocomotion. These in vivo data suggest that the activation of A14 dopamine cells in the PeVN of the hypothalamus is a cellular substrate for amphetamine to impact mobility, and their drive on the LS is independent of those of midbrain dopamine neurons^30,72,73^.

## Discussion

It is a formidable challenge to assess the functional complexity of the many hypothalamic nuclei and their emerging subdomains given the extraordinary cellular diversity in this brain region^1,16^. The introduction of single-cell profiling tools coupled with viral-assisted neurocircuit interrogation by now allows us to map molecular requirements of function determination in hypothalamic neurons at never-before-seen resolution^12,13,74–78^. Because of these technical advances, a paradigm shift is taking place with the hypothalamus being recognized, beyond its hormonal control of body-wide endocrine and metabolic processes, for its contribution to complex behaviors through extrahypothalamic projections, particularly towards limbic and motor systems^79–81^. Therefore, expanding knowledge on hypothalamic neurocircuit functions, output, and drug sensitivity is of paramount interest for translational and clinical biosciences.

Here, we focused on A14 neurons to identify the level of their heterogeneity by sophisticated electrophysiology, tract tracing, and behavioral assays. Our data allowed us to recognize neuronal subdivisions in the PeVN, an elongated nucleus that stretches along the wall of the 3rd ventricle over >2 mm in mice. We demonstrated that anterior A14 dopamine neurons are wired into the core circadian clock neurocircuit by revealing them being targeted by NMS^+^ excitatory inputs from the SCN. This assertion was additionally justified by diurnal variations in (i) NMS levels, (ii) the amount of biochemically active tyrosine hydroxylase (defined by Ser^40^ phosphorylation), and (iii) a significant increase in A14 neuron excitability during the night phase evoked by the increased frequency of spontaneous excitatory postsynaptic currents. These data support that A14 dopamine neurons undergo rhythmic cell-state changes, which do not only manifest as expressional oscillations for transcription factors and neuropeptides^41^ but the robustness of their excitability and synaptic output. The fact that A14 neurons are entrained by the SCN formulate the concept that it is their efferent output and postsynaptic target specificity rather than molecular determinants of intrinsic excitability that engrave network specificity and function assignment.

Among the many concepts of a hypothalamic organization that had prevailed for decades is the perception of neurochemical unity and continuity, assuming that the neurotransmitter phenotype of neurons alone is sufficient to define functions through the spatial segregation of efferent connectivity. For dopamine neurons, an increasing number of studies show that their subtypes instead differ in a matrix of parameters, including transcription factors, developmental trajectories, input-output constellations, and, for some, circadian clock features. It is therefore not surprising that the detailed interrogation of circuit maps for subsets of dopamine neurons by sophisticated experimental strategies^3,5,13,22,56^ lags 50 years behind their first anatomical description as the A11, A13, A14, A15 dopamine loci^20,21,82^. At present, brain-wide imaging is customary to identify hitherto unknown neuronal projections and to precisely dissect if intercalated cohorts of neurons in fact produce topologically organized afferentation maps^83,84^. Here, we worked at the intersection of immunohistochemistry, tissue-clearing, and intact brain imaging at the single axon level to describe anterior vs. posterior A14 dopamine contingents: anterior subtype “1” neurons project to the LS. In contrast, axons of posterior subtype “2” dopamine neurons preferentially terminate at the external layer of the ME, thus being classical neuroendocrine output cells. The existence of these subclasses is reminiscent of those for, e.g., magnocellular oxytocin and parvocellular corticotropin-releasing hormone-containing neurons^56,63,79,80^, even if in some cases collateral axons rather than spatially segregated cell pools may be sufficient to serve for either endocrine or neuronal functions. The positional and morphological dichotomy amongst dopamine neurons allowed us to assign their pool in the anterior PeVN to being hypothalamic relays of the clock network, periodically modulating the dorsolateral segment of the LS. Considering that the dorsolateral LS is positioned between upstream hippocampal spatial information processing and downstream movement regulation^28,51^ its entrainment by the circadian clock circuit integrates the element of time to define inactive vs. active daily periods, particularly in the nocturnal species studied here.

Our ex vivo Ca^2+^ imaging experiments showed that pharmacogenetically activating nerve terminals of A14 dopamine neurons leads to global, synchronous, and protracted network activity in the LS. This effect was mediated by dopamine action on metabotropic D_1_ and D_2_ receptors postsynaptically, whose cellular diversity defined activation vs. inhibition through G_s_ and G_i_ protein recruitment, respectively^59,62^. Nevertheless, hypothalamic dopamine cells differentiate from a prototypic GABA progenitor phenotype of all during brain development^40^ and retain *Gad1/2* and *Slc32a1* expression of in adulthood. To this end, we tested the hypothesis that GABA could contribute to neuromodulation in the LS either through disinhibition of *Sst*^+^ neurons or directly. In contrast to other hypothalamic dopamine groups^56,65^, we did not find a biologically meaningful contribution of GABA signaling at either the neuronal network or behavioral levels, emphasizing the purely dopaminergic mode of operation for A14 neurons. We suggest that a GABAergic molecular phenotype might be used either in specific signaling contexts or recruited to specific projections dominating, e.g., intrahypothalamic connectivity and/or contributing to dendritic (retrograde) neurotransmission described for neurons^85^.

**Fig. 7:**
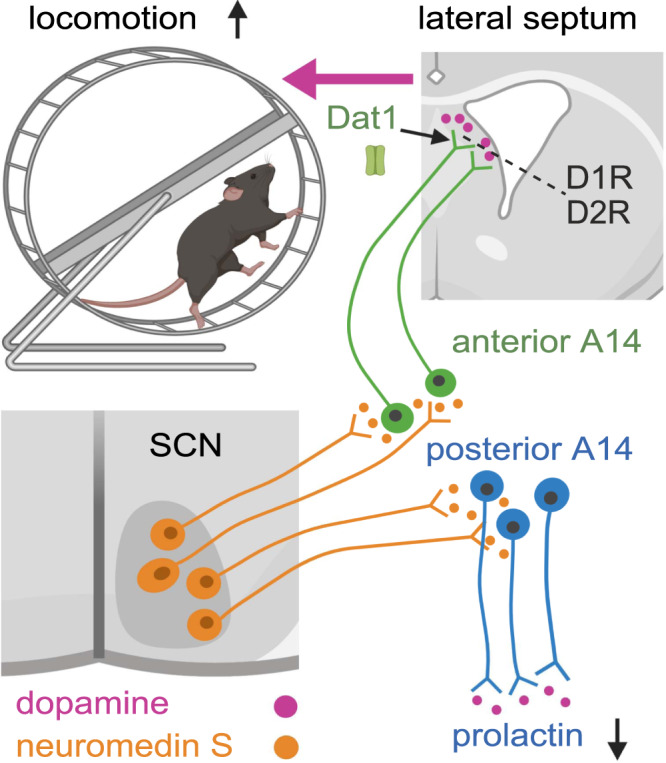
Illustration summarizing and contextualizing the experimental findings. Biorender assisted with our graphical design.

At the organismal level, we found that chemogenetic activation of A14 dopamine neurons of the anterior PeVN led to hyperlocomotion in the active phase of their circadian cycle, suggesting that this neuronal ensemble chronospecifically modulates animal activity. Based on our experimental data, we can suggest that neurotransmitter levels and content fluctuate in the A14-to-LS pathway during the circadian cycle. This notion is experimentally supported by the periodic increase in the enzymatically active (that is, phosphorylated) form of tyrosine hydroxylase only during the dark period. Similar molecular regulatory mechanisms (e.g., phosphorylation) can equally impact receptor sensitivity and competence for signal transduction in LS neurons. Along these thoughts, our results significantly go beyond existing knowledge not only on the synaptic layout and hierarchy of the SCN → anterior PeVN → LS circuit for the circadian control of locomotion^28,29,48,66^ but regarding cellular substrate(s) for psychostimulant action. Psychostimulant effects in the brain are, in a large part (but see ref. 86), mediated by inhibition of *Dat1* on neurons^87^. Direct DAT inhibition increases extracellular dopamine availability, thus causing excess excitation that evokes hyperlocomotion. The explanation provided here on how amphetamine can override the neural control of the central pacemaker on locomotion is of significant human relevance because shift workers, combat pilots, and others extensively use amphetamine to increase focus and boost their own activity at night, which is the traditionally passive phase in humans. We also propose that increased evening and night activity, a primary side effect of methylphenidate treatment for attention deficit hyperactivity disorder (ADHD), might also be related to the drug’s efficacy to inhibit both dopamine and norepinephrine reuptake along the SCN → anterior PeVN → LS circuit. Altogether, our results identify A14 dopamine neurons as a hypothalamic junction to link the hypothalamus to executive neurocircuits of a motivated behavior in a circuit design that complements and expands the well-described role of midbrain dopamine neurons in locomotion^88^ (Fig. 7).

## Methods

### Animals

Mice were housed under a 12/12-h light/dark cycle (CT 00:00—light on; CT 12:00—light off) in a temperature (22 ± 2 °C) and humidity (50 ± 10%) controlled environment with food and water available ad libitum. We used Tg(Th-EGFP)21-31Koba (Riken Bioresource Center)^13^ and *Dat1*-Ires-Cre (JAX:006660)^89^ mice, the latter crossed with Ai14 (JAX:007914)^90^ reporters for the anatomical and electrophysiological characterization of A14 neurons. Homozygous *Dat1*-Ires-Cre mice were used for the viral targeting of A14 neurons with Cre-dependent AAV viruses (Supplementary Table 1). Data for retrograde axonal tracings were obtained from wild-type C57Bl/6J (Charles River) males. *Drd1*-GFP and *Drd2*-GFP^52,53^ (GENSAT, Rockefeller University) mice were used to validate D_1_/D_2_ receptor expression in *Sst*^+^ neurons of the LS. No statistical methods were used to predetermine the sample size. The experiments were not randomized and did not require data collection in a blinded fashion. Tissue collection and experiments on live animals conformed to the 2010/63/EU directive and were approved by the Austrian Ministry of Science and Research (66.009/0277-WF/V/3b/2017). The 3Rs (Replacement, Reduction, and Refinement) were systematically applied throughout the study. Particular effort was taken to minimize animal suffering during experiments.

### Virus microinjections in vivo

Stereotaxic injections were performed as described earlier^13,63^. Briefly, mice were anesthetized with isoflurane (5%, 1 L/min flow rate) and placed in a stereotaxic frame (RWD). A Stereotaxic Injector (Stoelting or RWD) was used to inject virus particles at a speed of 50 nl/min. A 5-min delay was used prior to withdrawing the glass pipette (Drummond) to achieve optimal and localized viral diffusion in situ. Viral constructs were listed in Supplementary Table 1. Fourteen to 21 days after injection, animals were used for (i) mapping local and long‐range projections, (ii) ex vivo optogenetics, (iii) Ca^2+^ imaging, and (iv) behavioral tests. For chemo- and optogenetic stimulation, Cre-dependent viruses were bilaterally injected into the PeVN of *Dat1*-Ires‐Cre mice (40–50 nl/side; coordinates relative to bregma: AP: −0.6 mm, DV: 5.15 mm, ML: ±0.2 mm). Cre-independent AAVs carrying DREADDs were bilaterally injected into the LS (75 nl/side, coordinates relative to bregma: AP: +0.26 mm, DV: 3.25 mm, ML: ± 0.5 mm) alone or five days after delivering a Cre-dependent viral construct into the PeVN (for the simultaneous modulation of the PeVN and LS). In Ca^2+^ imaging experiments, viruses containing activating DREADD (hM3Dq) were bilaterally expressed in A14 dopamine neurons (40 nl/side; coordinates relative to bregma: AP: −0.6 mm, DV: 5.15 mm, ML: ±0.2 mm). Three days later, Cre-independent AAVs carrying GCAMP6f were bilaterally injected into the LS (75 nl, coordinates relative to bregma: AP: +0.26 mm, DV: 3.25 mm, ML: ±0.5 mm).

### Anterograde tracing

*Dat1*-Ires-Cre mice (*n* = 13 at 3 weeks after birth) received a unilateral injection of either AAV-hSyn-DIO-mCherry or AAV-hSyn-DIO-EGFP.WPRE.hGH virus at two AP positions within the PeVN (40 nl; coordinates from bregma: (i) AP: −0.6 mm, DV: 5.15 mm, ML: ±0.2 mm or (ii) AP: −1.6 mm, DV: 5.10 mm, ML: ±0.2 mm) under deep anesthesia (see above) with their heads fixed in a stereotaxic frame. Fourteen to 25 days after surgery, the animals were transcardially perfused, and their brains were processed for immunohistochemistry or light-sheet microscopy.

### Retrograde tracing

C57Bl/6J mice (*n* = 3 at 3 weeks of age) received a unilateral injection of retrograde AAV-CAG-tdTomato (75 nl) tracer to the LS at coordinates: AP: +0.26 mm, DV: 3.25 mm, ML: ±0.5 mm or AP: 0.5 mm, DV: 2.5 mm, and ML: −4.5 mm. After a 21‐day survival period, animals were transcardially perfused, with their brains processed for immunohistochemistry.

### Immunohistochemistry

Animals were anesthetized with phenobarbital (50 mg/ml, Sigma BP901) and transcardially perfused with a fixative composed of 4% paraformaldehyde (PFA) in 0.1 M phosphate buffer (PB; pH 7.4) that was preceded by a short pre-rinse with physiological saline containing heparin (10 units/ml, Sigma H0777). In experiments investigating diurnal changes in TH phosphorylation and neuromedin-S production, transcardial perfusions were carried out during the periods of CT: 08:00–09:00 (day, light) and 20:00–21:00 (night, dark). After overnight postfixation in the same fixative, 50 μm-thick serial free-floating sections were coronally cut on a vibratome (Leica VT1200S) and processed for multiple immunofluorescence histochemistry. The list of primary antibodies used for the study is provided in Supplementary Table 2. mCherry and TdTomato immunofluorescence were amplified in virally infected and transgenic animals using polyclonal anti-mCherry antibody (EnCor Biotech). Non-specific immunoreactivity was suppressed by incubating free-floating sections in a blocking cocktail composed of 5% normal donkey serum (NDS; Jackson), 2% bovine serum albumin (BSA; Sigma), and 0.3% Triton X-100 (Sigma) in PB for 1 h at 22–24 °C. Sections were then exposed to a specific combination of primary antibodies in PB also containing 2% NDS, 0.1% BSA, and 0.3% Triton X-100 at 4 °C for 48–72 h.

For post-hoc analysis, 300 μm-thick vibroslices after patch-clamp recordings were immersion fixed in 4% PFA in 0.1 M PB at 4 °C for 18 h. Next, sections were thoroughly washed with PB and processed to suppress non-specific immunoreactivity in 0.1 M PB to which 10% NDS, 5% BSA and 0.5% Triton X-100 had been added for 2 h. Subsequently, slices were immersed into a primary antibody solution that consisted of 2% NDS, 0.1% BSA, 0.5% Triton X-100, and 5% DMSO (Sigma) in PB for 72 h (Supplementary Table 2).

Immunoreactivities were revealed by Alexa Fluor 488, carbocyanine (Cy) 3; Alexa Fluor 488, or Cy5-tagged secondary antibodies diluted in 2% BSA in PB (1:300, Jackson ImmunoResearch) at 22–24 °C for 2 h. To visualize cells filled with biocytin, AlexaFluor-conjugated streptavidin (1:300, Jackson ImmunoResearch) was used in a mixture of secondary antibodies. Sections were mounted on fluorescence-free glass slides and air dried for 30 min prior to being coverslipped with Entellan (in toluene; Merck).

### Laser-scanning microscopy and image analysis

Images were acquired on a Zeiss LSM 880 laser-scanning microscope at ×20, ×40, or ×63 primary magnification. The emission spectrum for each dye was limited as follows: Alexa488/Cy2 (505–530 nm), Cy3 (560–610 nm), and Alexa647/Cy5 (650–720 nm). Biocytin-filled neurons were 3D‐reconstructed from *z*-stack images acquired at ×40 primary magnification using the ZEN2013 software package (Zeiss). For the analysis of phospho^40^-TH and TH immunofluorescence, montages of confocal micrographs in single optical sections spanning the entire PeVN were acquired at ×20 or ×10 primary magnification. Three serial sections/animal were selected for quantification of fluorescently labeled neurons. The PeVN was divided into three levels: *rostral*, bregma, approx. −0.2 mm; *mid*, bregma −0.6 mm (at the suprachiasmatic nucleus); and *caudal*, bregma −0.9 mm (at the retrochiasmatic nucleus). All images were acquired with the same laser power, pinhole, master, and digital gain settings on an LSM 880 microscope. Quantitative analysis of the immunofluorescence intensity was performed in Fiji ImageJ for the individual color channels. To determine diurnal changes in neuromedin-S expression, labeled neurons were inspected in the SCN. Two sections/animal were selected for quantification of the fluorescence intensity using the ZEN2013 software.

### Electron microscopy

Animals were transcardially perfused with 4% PFA and 0.1% glutaraldehyde in 0.1 M PB. Brains were dissected out and cut on a vibratome (Leica V1200S, 50 µm). Sections were processed for TH immunohistochemistry (pre-embedding chromogenic detection with mouse anti-TH (1:500; Millipore MAB5280, clone 2/40/15) and biotin-SP (long spacer) AffiniPure Donkey Anti-Mouse IgG (1:1000; Jackson ImmunoResearch Cat. № 715-065-151), then post-fixed in buffered 1% OsO_4_ at 22–24 °C for 1 h, and flat-embedded in Durcupan ACM (Fluka)^63^. The LS was selected and embedded for ultrasectioning. Ultrathin sections (100 nm) were collected on single-slot nickel grids coated with Formvar and subsequently processed for post-embedding VGAT-immunogold staining. The primary anti-VGAT antibody (SySy, rabbit, Cat. no. 131 003) was diluted 1:100. Anti-rabbit IgG-coated colloidal gold antibody (BBI Solutions, Cat. no. EM.GAR15) was diluted 1:15.

### Whole-brain tissue clearing and light-sheet microscopy

*Dat1*-Ires-Cre mice underwent unilateral injection of AAV-hSyn-DIO-EGFP.WPRE.hGH construct into the PeVN. Three weeks after the injection, the animals were transcardially perfused, and their brains dissected for optical clearing with a dehydration-based approach.

Cleared samples were illuminated with the light sheets of an ultramicroscope from two sides^45^ with either of the two Sapphire lasers: 488 nm/200 mW and 532 nm/200 mW (Coherent, Germany). An Andor Neo 2560 × 2160 camera with 6.5 µm pixel size was used for image capture. Two filters were used for image processing: 525/20 ET Bandpass F47-528 (AHF, Germany) and 605/70 ET Bandpass F47-605 (AHF, Germany). Signal detection refractive index (RI)-corrected objectives were used: (i) ×4 objective (Olympus, XLFluor4×/340, 0.28 NA, WD = 29.5 mm) utilizing custom-built correction of optics for a RI of 1.56 (WD = 10 mm post correction) and (ii) ×16 objective (Leica, HC FLUOTAR L ×16 NA, 0.6 IMM CORR, WD = 2.5 mm) adjusted to an RI of 1.56. Imaging specifications for individual panels, including objectives, pixel size, z-step, imaging depth, and orientation, from which the image was recorded, are provided in Supplementary Table 3. Images were deconvolved as reported earlier^91^. Briefly, image processing from light-sheet microscopy was performed by using MATLAB 9 (MathWorks) with contrast-limited adaptive histogram equalization (CLAHE) and Fast-Fourier-Transform-based de-striping algorithms^91^. Amira 6.7 software (Thermofisher) was used for 3D image reconstruction.

### RNA-scope in situ hybridization

*Dat1*-Ires-Cre mice containing Cre-dependent viral tracer(s) and *Drd1*-EGFP/*Drd2*-EGFP mice were used to verify potential targets of A14 efferent projections in the LS and to assess whether innervated neurons expressed D1/D2 receptors. Mice were transcardially perfused with 4% PFA in PB (pH 7.4). After overnight postfixation of the brains in the same fixative, samples were incubated in 30% sucrose for at least 48 h and then cryostat-sectioned at 20-μm-thickness directly onto fluorescence-free glass slides. RNA-scope 2.0 was performed according to the manufacturer’s instructions (Advanced Cell Diagnostics)^92^. RNA-scope probes for the detection of *Sst* ad *Egfp* were designed by the manufacturer and are commercially available. Imaging was performed using A Zeiss LSM 880 microscope equipped with a ×40 objective.

### Ca^2+^ imaging

Coronal slices containing the LS were prepared from *Dat1*-Ires-Cre mice (4-5 weeks old) after AAV-hSyn-DIO-hM3D(Gq)-mCherry or sham injections to the PeVN and subsequent injection of AAV-hSyn1-GCaMP6f-P2A-nls-dTomato into the LS. 2–3 weeks after viral injections, mice were deeply anesthetized (phenobarbital, 50 mg/ml) and perfused with ice-cold preoxygenated (95% O_2_/5% CO_2_) cutting solution containing (in mM) 93 N-methyl-d-glucamine (NMDG), 30 NaHCO_3_, 2.5 KCl, 1.2 NaH_2_PO_4_, 20 HEPES–NaOH, 5 Na-ascorbate, 3 Na-pyruvate, 0.5 CaCl_2_, 8 MgSO_4_, and 25 glucose, pH 7.4^93^. Next, 300 μm-thick coronal slices were cut on a vibratome (VT1200S, Leica). Slices were then transferred to a recovery chamber filled with the same solution (32 °C) for 12 min and kept (a minimum 60 min prior to the recordings) in a solution containing (in mM): 90 NaCl, 26 NaHCO_3_, 3 KCl, 1.2 NaH_2_PO_4_, 20 HEPES–NaOH, 5 Na‐ascorbate, 3 Na‐pyruvate, 1.5 CaCl_2_, 2 MgSO_4_, 0.5 l‐glutathione, and 25 glucose (pH 7.4, 22–24 °C). Recordings were performed in oxygenated (95% O_2_/5% CO_2_) artificial cerebrospinal fluid (ACSF) containing (in mM): 126 NaCl, 2.5 KCl, 1.25 NaH_2_PO_4_, 26 NaHCO_3_, 2 MgSO_4_, 1.5 CaCl_2_, and 12.5 glucose (pH 7.4, 22–24 °C). Ca^2+^ measurements were done using a VisiChrome monochromator to excite GCaMP6f and VisiView 3.0.3.0 software (Visitron Systems) on an AxioExaminer.D1 microscope (Zeiss) equipped with a CoolSnap HQ2 camera (Photometrics) and a water‐immersion ×20/differential interference contrast objective (Plan‐Apochromat/N.A. 1.0). To infer diurnal changes in basal A14 neuronal activity, animals were processed for Ca^2+^ measurements during the day (slice preparation started at CT: 04:00; recording during CT: 06:00-11:00) and night (slice preparation started at CT:16:00 with recording during CT:18:00-23:00). DREADDs were stimulated by superfusing clozapine N-oxide dihydrochloride (20 µM, CNO, Tocris Cat. No. 6329). R(+)-SCH-23390 (1 µM, Sigma D054) and (±)-sulpiride (10 µM, Sigma S8010) were used to block D1 and D2 receptors, respectively. Drugs were directly applied into the recording chamber at the final concentration indicated. Ca^2+^ responses were evaluated using Clampfit 10.0 (Molecular Devices) and GraphPad Prism 7 (GraphPad).

### Patch-clamp electrophysiology and ex vivo optogenetics

Whole-cell recordings were carried out as described^63,94^. *Dat1*-Ires-Cre mice crossed with an appropriate reporter strain or expressing a Cre-dependent virus in the PeVN (*n* = 38; 21–28 days old) and TH-GFP mice (*n* = 5; 21–28 days old) were used for patch-clamp recordings. Mice were deeply anesthetized and perfused with ice-cold pre-oxygenated cutting solution (see the section “Ca^2+^ imaging”). Subsequently, 300 μm‐thick coronal slices were cut on a vibratome with a pre-selection of slices containing the PeVN. For optogenetic stimulation, slices containing the LS were selected. After a 12-min recovery period at 32 °C, all slices were immersed in ACSF at 22–24 °C for 1–5 h before recording. For the experiments involving measurements of diurnal changes in the intrinsic properties of A14 DAT^+^ neurons, mice were perfused and used for electrophysiological recordings during the day (start of slice preparation at CT 04:00; recording during CT:06:00–11:00) and night (start of slice preparation at CT 16:00; recording during CT:18:00–23:00). Whole‐cell recordings in current‐clamp or voltage‐clamp mode were carried out on an EPC-10 triple amplifier (HEKA) controlled by PatchMaster 2.80. Glass electrodes of 3–5 MΩ resistance were filled with an intracellular solution containing (in mM): 130 K-gluconate, 3 KCl, 4 ATP-Na_2_, 0.35 GTP-Na_2_, 8 phosphocreatine-Na_2_, 10 HEPES, 0.5 ethyleneglycol-bis(2-aminoethylether)-*N*,*N*,*N*′*,N*′*-*tetraacetate (EGTA), (pH 7.2 set with KOH) and 0.5% biocytin (Sigma) to allow for post-hoc neuronal reconstruction. Base current and spontaneous excitatory postsynaptic currents (EPSCs) were recorded from DAT^+^/TH^+^ neurons clamped at −70 mV. To investigate the sensitivity of A14 neurons to neuromedin-S, recombinant peptide (Tocris Cat. No. 3648) was administrated directly into the bath chamber at a final concentration of 1 µM.

Ex vivo optogenetics employed excitation switches between 470 and 535 nm on a pE‐100 CoolLED illumination system (CoolLED) at a uniform light intensity of 0.2 mW, measured at the tissue surface. AAV-EF1a-double floxed-hChR2(H134R)-mCherry-WPRE-HGHpA virus was bilaterally expressed in 3-week-old *Dat1*-Ires-Cre mice (*n* = 24) at the PeVN. Two to three weeks after injection, animals were used for electrophysiological recordings as described above. Light stimulation was corrected for light intensity and duration to achieve optimal excitation of ChR2 terminals in the LS. Electrophysiological data were analyzed using Clampfit 10.0 (Molecular Devices) and GraphPad Prism 7 (GraphPad).

### Monitoring of home-cage activity

To investigate the role of A14 neurons and their neurotransmitter systems in defining locomotor activity, we used different viruses (Fig. 5a, Supplementary Fig. 7e) including negative controls (Supplementary Fig. 7c, d). Seventeen days after the last surgery, animals were single housed in PhenoTyper cages (Noldus) with one day of habituation and 6 days of continuous activity measurements. Food and water were available ad libitum. DREADDs were stimulated by diluting CNO in the drinking water (5 mg/kg, calculated upon average water intake being 5 ml per day) with 2–3 experimental repeats for each animal. Home-cage activity was analyzed from CT 00:00–06:00 (“day-time”) and CT 12:00–18:00 (“nighttime”). The following parameters were defined: distance moved (m), total activity (%), walking duration (%), and wheel running (number of wheel rotations). At the end of the experiments, the animals were sacrificed, with their brains used for *post-hoc* verification of the injection sites. Data were analyzed using EthoVision XT15 software (Noldus) and GraphPad Prism 7 (GraphPad).

### Home-cage activity monitoring before and after amphetamine exposure

*Dat1*-Ires-Cre mice (3 week-old, *n* = 7) received a bilateral injection of AAV-hSyn-DIO-hM4D(Gi)-mCherry virus^95^ into the PeVN. After 21 days of recovery, animals were single-housed in PhenoTyper cages (Noldus) with a 1-day habituation period prior to home cage activity monitoring with food and water available ad libitum. DREADDs were activated via CNO administration in the drinking water (5 mg/kg, calculated based on 5 ml/day average water intake). Measurements of motor activity were performed for 2 days (12 h day/12 h night) using normal drinking water to determine their baseline activity, followed by 2 days of CNO treatment. To estimate the effects of dopamine signaling in the PeVN on the animal motor activity, the same mice were administered intraperitoneal amphetamine (10 mg/kg, Sigma) after a day of post-CNO recovery. Amphetamine was administrated at CT 00:00 and 12:00 for 4 days to determine changes in diurnal activity in the absence (2 days) or presence (2 days) of CNO in the drinking water. The sequence of single amphetamine and amphetamine + CNO treatment alternated between animal batches. The activity was evaluated from 6-h epochs of monitoring during the day (CT 00:00–06:00) and night (CT 12:00–18:00) by the following parameters: distance moved, walking duration (%), total activity (%), and wheel running (number of wheel rotations). After the experiments, animals were sacrificed, and their brains were used for post-hoc histochemical analysis. Data were analyzed using the EthoVision XT15 software (Noldus) and GraphPad Prism 7 (GraphPad).

### Statistics and data analysis

None of the experiments required data collection in a blinded fashion. Fold changes represent the percentage change from the mean control value in individual experiments. A *p*-value of <0.05 was considered statistically significant. Quantitative analysis of immunoreactive neurons and immunofluorescence intensities were evaluated by unpaired Student’s *t*-test. Data from Ca^2+^‐imaging experiments and electrophysiology studies were defined by unpaired or paired two-tailed Student’s *t*-test when appropriate. Behavioral data upon chemogenetic manipulation of A14 and LS neurons, as well as after amphetamine exposure were statistically assessed by paired Student’s *t*-test or ANOVA. Statistical assessment was undertaken in either GraphPad Prism 7 or SigmaPlot 13.0. Gene expression in single-cell RNA-seq count matrices from refs. 13, 40 were analyzed by basic functions of the Seurat3^96^ (https://satijalab.org/seurat/articles/pbmc3k_tutorial.html) and tidyverse^97^ packages.

### Figure preparation

Multi‐panel figures were assembled in CorelDraw X9 (Corel Corp.). We used Biorender and Adobe Illustrator CS6 to draw schemes and illustrations. Photoshop CS6 (Adobe) was used to crop the original histochemical images.

### Reporting summary

Further information on research design is available in the Nature Research Reporting Summary linked to this article.

## Supplementary information

Supplementary Information

Description of Additional Supplementary Files

Supplementary Movie 1

Supplementary Movie 2

Reporting Summary

## Data Availability

Single cell RNA-seq data were published earlier and can be downloaded in raw and processed forms from NCBI’s Gene Expression Omnibus with accession numbers GSE74672 and GSE132730. Data generated in this study are available in the Source Data file. Source data are provided with this paper.

## Source data

Source Data

## Code availability

The code used for data analysis can be found in the following GitHub repository: https://github.com/Harkany-Lab/Korchynska22_Nature_Comm
