# Supplementary material for: A hypothalamic dopamine locus for psychostimulant-induced hyperlocomotion in mice
Source: Nat Commun. 2022 Oct 8;13:5944. doi: 10.1038/s41467-022-33584-3 (PMC9547883; doi:10.1038/s41467-022-33584-3)
Supplement: Supplementary file 1 — Supplementary Information [file 41467_2022_33584_MOESM1_ESM.pdf]

Supplementary information file for the manuscript:

A hypothalamic dopamine locus for psychostimulant-induced hyperlocomotion in mice

Solomiia Korchynska, Patrick Rebernik, Marko Pende, Laura Boi, Alán Alpár, Ramon Tasan, Klaus Becker, Kira Balueva, Saiedeh Saghafi, Peer Wulff, Tamas L. Horvath, Gilberto Fisone, Hans-Ulrich Dodt, Tomas Hökfelt, Tibor Harkany and Roman A. Romanov

Address for correspondence: Dr. Tibor HARKANY (Tibor.Harkany@meduniwien.ac.at), or Dr. Roman A. ROMANOV (roman.romanov@meduniwien.ac.at); both at the Medical University of Vienna

This file contains:

Supplementary figures 1-7

Legends to supplementary figures

Supplementary tables 1-3

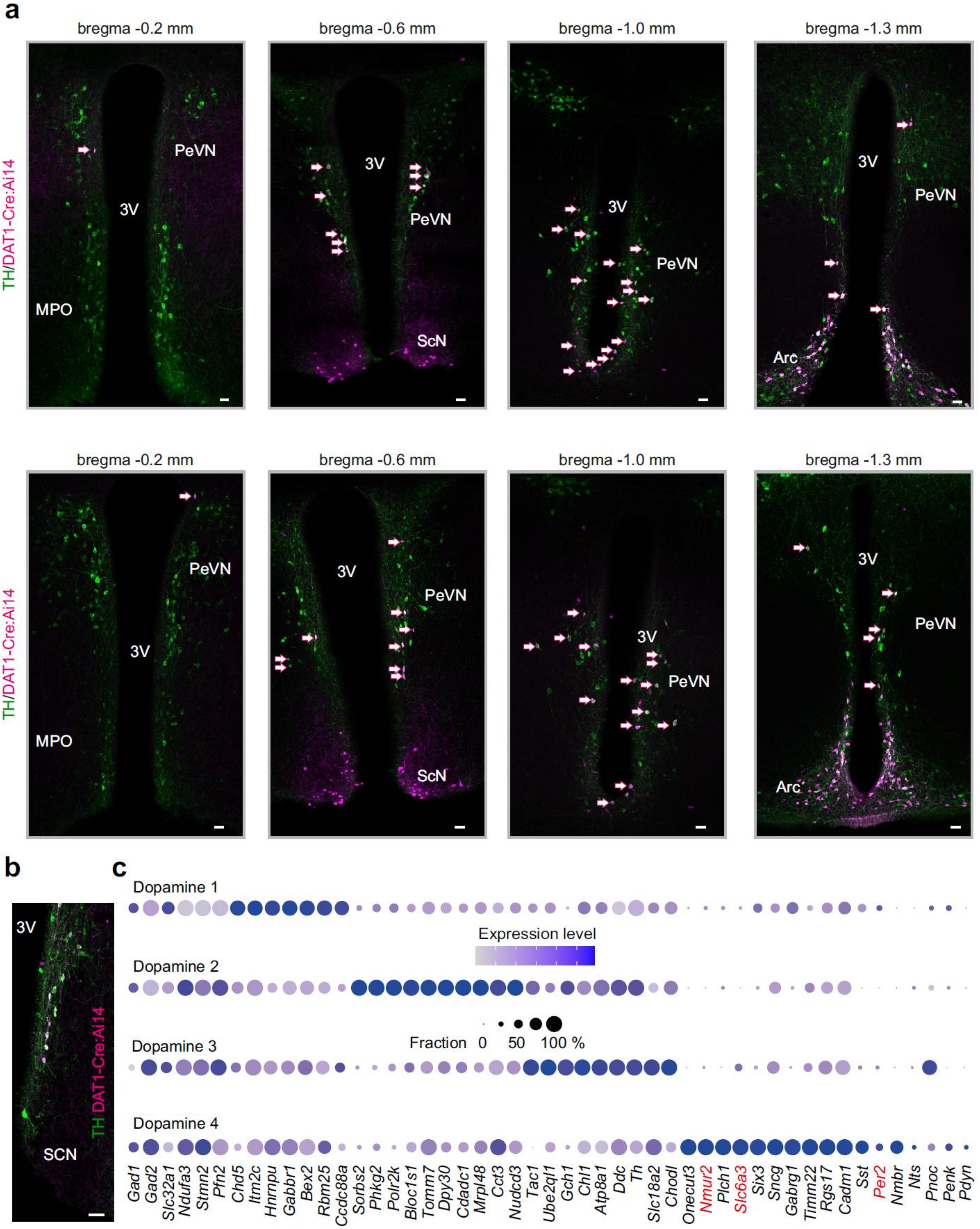
a
bregma -0.2 mm
bregma -0.6 mm
bregma -1.0 mm
bregma -1.3 mm
TH/DAT1-Cre:Ai14
PeVN
3V
MPO
ScN
Arc
b
3V
SCN
TH DAT1-Cre:Ai14
c
Dopamine 1
Dopamine 2
Dopamine 3
Dopamine 4
Expression level
Fraction 0 50 100 %
Gad1
Gad2
Slc32a1
Ndufa3
Stmn2
Pfn2
Chd5
Itm2c
Hnrnpu
Gabbr1
Bex2
Rbm25
Ccdc88a
Sorbs2
Phkg2
Polr2k
Bloc1s1
Tomm7
Dpy30
Cdadc1
Mrpl48
Cct3
Nudcd3
Tac1
Ube2ql1
Gch1
Chl1
Atp8a1
Ddc
Th
Slc18a2
Chodl
Onecut3
Nmur2
Plch1
Slc6a3
Six3
Sncg
Gabrg1
Timm22
Rgs17
Cadm1
Sst
Per2
Nmbr
Nts
Pnoc
Penk
Pdyn

Supplementary Fig. 1. Anatomical mapping and molecular characterization of *Dat1*⁺ dopamine neurons with a focus on the PeVN of the hypothalamus. a, Rostrocaudal distribution of *Dat1*⁺ neurons co-labelled for TH (in green) in *Dat1*-Ires-Cre:Ai14 reporter mice (Ai14 was color-coded in magenta). Images from two representative animals are shown (top *vs.* bottom rows). Scale bars = 50 μm. **b,** Low-power survey for *Dat1*⁺ A14 dopamine neurons co-expressing TH and tdTomato (marked by *arrows*) in the PeVN of *Dat1*-Ires-Cre:Ai14 mice. Scale bar = 50 μm. **c,** Single-cell RNA-seq data reprocessed from an open-label dataset[13] demonstrates differentially-expressed genes among the dopamine neuron subtypes that had so far been identified. The "dopamine-4" group corresponds to A14/PeVN neurons and differs in its expression of key genes, including transcription factors (*Onecut3*), dopamine transporter (*Slc6a3)*, neuropeptide receptors (*Nmur2, Nmbr)*, and clock genes (*Per2*). The experiment shown in (**a**) and (**b**) was independently repeated 9 times with similar results.

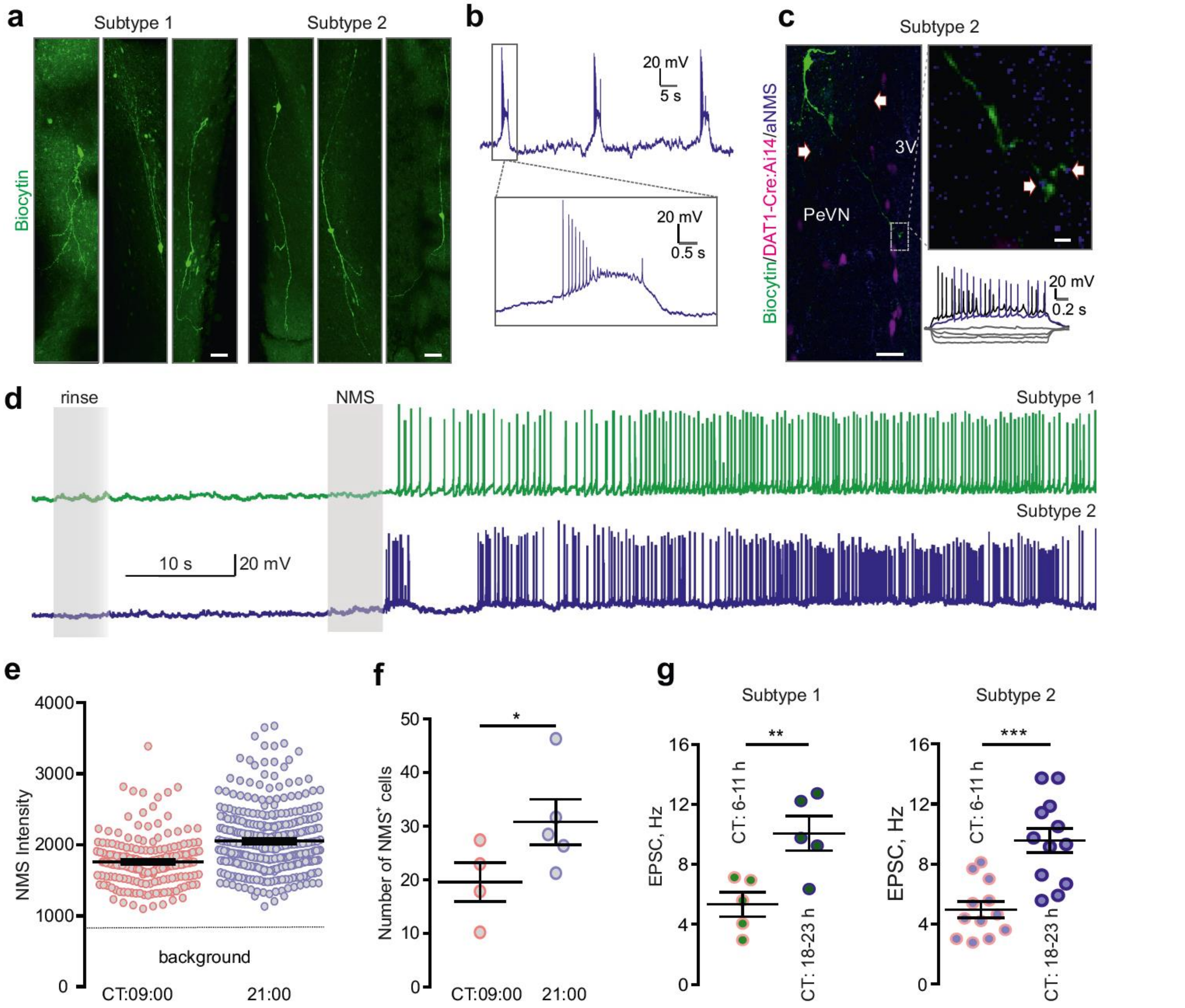

Supplementary Fig. 2. Morpho-physiological characteristics of Dat1+ and NMS+ hypothalamic neurons. **a**, Biocytin-filled Dat1+ neurons of the PeVN. Three cells are shown, each for a separate subtype. Scale bars = 50 μm. **b**, A typical current-clamp recording of spontaneous firing activity in a subtype "2" neuron. Open rectangle indicates a single burst shown at high temporal resolution. **c**, Post-hoc immunohistochemical detection of neuromedin S (NMS) in putative synaptic terminals apposing dopamine neurons of the PeVN. Low and high-resolution images show the presence of NMS in varicose structures in close contact to biocytin-filled dopamine neurons (arrows). Open rectangle denotes the location of the insets. Representative AP signature identifies a subtype "2" neuron. The experiment was independently repeated 3 times with similar results. Scale bars = 50 (left) and 10 μm (right). **d**, Representative current-clamp recordings of NMS-induced AP firing (see also Fig. 1f) for the two identified neuronal subtypes excluding mechanical handling as a factor to either bias or evoke the electrophysiological responses. **e**, NMS fluorescence intensity in the SCN at two time points (n = 4 animals, 11 unilateral slices for each experimental condition). The number of cells with detectable NMS expression was higher at 03:00 than at 15:00 (345 vs. 190 cells). Similarly, the average fluorescent intensity at 03:00 exceeded than at 15:00 (1991 vs. 1648 intensity units). No statistical test was applied to the latter parameter. Error bars correspond to mean ± SEM. **f**, Statistical analysis of the cell numbers with detectable NMS immunoreactivity in the SCN at two time points (data are presented as mean ± SEM, n = 4 and n = 5 animals respectively; p = 0.0471 when a one-sided unpaired t-test was applied; * - p < 0.05). **g**, Excitatory postsynaptic currents recorded in Dat1+ cells in the PeVN. Data were presented separately for the two identified subtypes of Dat1+ neurons. Data are presented as mean ± SEM, and statistical significance was estimated with unpaired two-sided t-test. ** - p < 0.01 (p = 0.0098); *** p < 0.001 (p < 0.0001). For subtype 1, n = 5 cells from 3 independent experiments (animals) for CT: 6-11 h, n = 5 cells from 3 independent experiments for CT: 18-23 h. For subtype 2, n = 12 cells for CT: 6-11 h from 3 independent experiments, n = 12 cells for CT: 18-23 h from 3 independent experiments. Abbreviation: 3V, third ventricle.

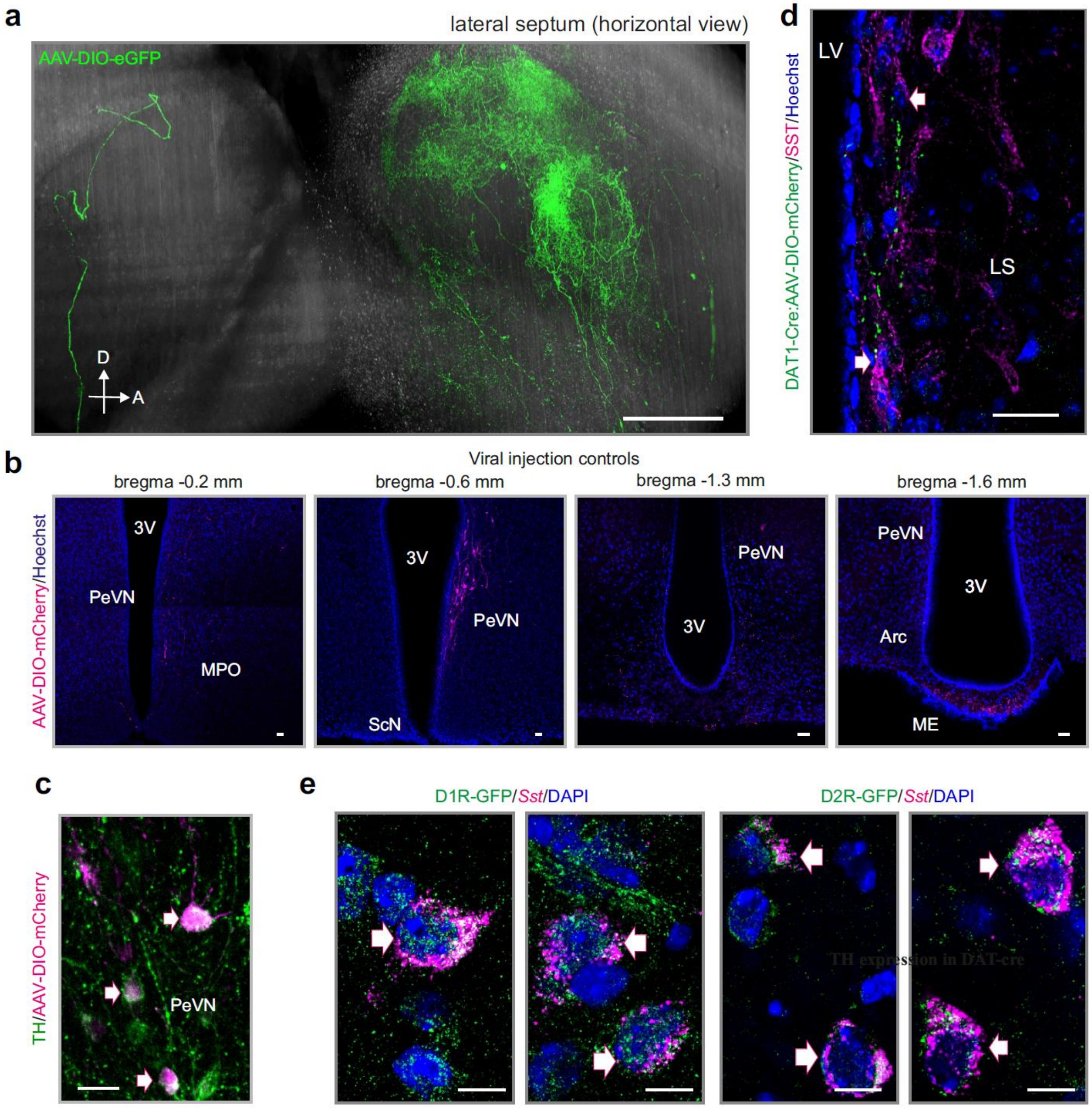

Supplementary Fig. 3. Extrahypothalamic targets of *Dat1*$^{+}$ neurons of the PeVN. a, Horizontal view of PeVN projections to the LS by light-sheet microscopy. The experiment was independently repeated 4 times with similar results. Scale bar = 200 μm. **b,** Serial images of coronal sections with a focus on the region adjacent to the 3rd ventricle to show the distribution of neurons with Cre-dependent production of mCherry as a fluorescent reporter. Forty nls of AAV-DIO-mCherry were injected into the PeVN of *Dat1*-Cre mice (bregma: AP: −0.6 mm, DV: 5.15 mm, ML: ±0.2 mm). Infected neurons were found concentrated around the site of the microinjection and but not in the MPO, posterior PeVN, and ARC. The experiment was independently repeated 12 times with similar results. Scale bar = 50 μm. **c**, Representative immunohistochemical images of cells coincidently positive for TH and mCherry that had produced viral particles (as in **b**) confirmed that *Dat1*$^{+}$ neurons of the anterior PeVN express TH. The experiment was independently repeated 9 times with similar results. Scale bar = 20 μm. **d,** Immunohistochemistry for SST and mCherry, the latter labelling neuronal projections from virus-infected *Dat1*$^{+}$ neurons in the LS. AAV-DIO-mCherry constructs were injected into the anterior PeVN area of *Dat1*-Cre mice. The experiment was independently repeated 3 times with similar results. Scale bar = 50 μm. **e,** I*n situ* hybridization for *Sst* combined with the immunohistochemical detection of either D1R-GFP (*left*) or D2R-GFP (*right*) in the LS. The experiment was independently repeated 2 times with similar results. *Abbreviations*: LV, lateral ventricle. Scale bars = 10 μm.

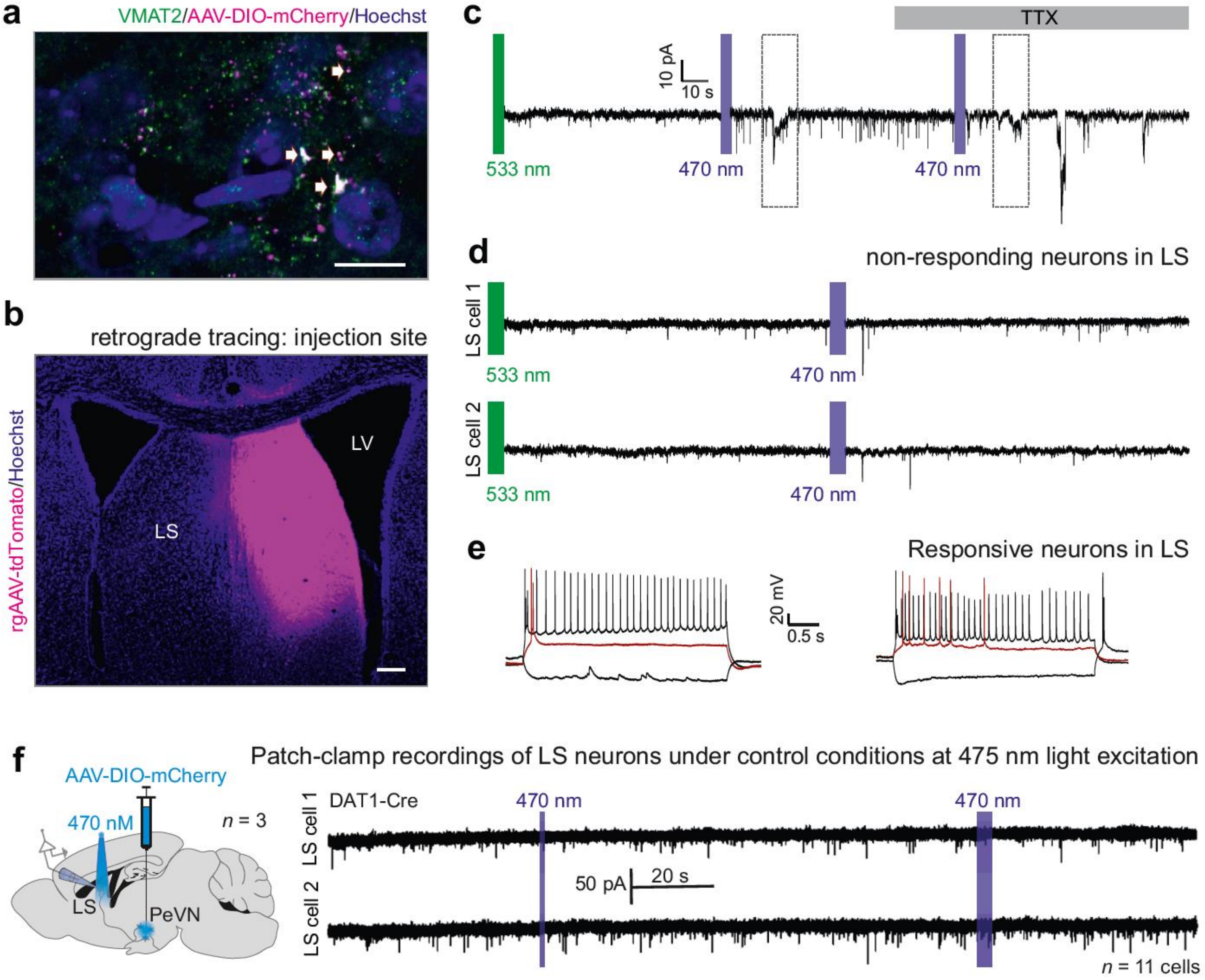

Supplementary Fig. 4. *Dat1*⁺ neurons of the PeVN innervate the LS. a, Immunohistochemistry showing the co-localization of VMAT2 and mCherry in projections of *Dat1*⁺ neurons in the LS. Samples were obtained from *Dat1*-IRES-Cre mice after stereotaxic injection of AAV particles to produce mCherry under the control of Cre-recombinase into PeVN. The experiment was independently repeated 3 times with similar results. Scale bar = 10 μm. **b,** Representative image showing tdTomato fluorescence in the LS at the site of rgAAV-tdTomato infusion applied as retrograde tracer. The experiment was independently repeated 3 times with similar results. Scale bar = 200 μm. **c,** Representative voltage-clamp recording demonstrating that tetrodotoxin (TTX) does not block inward transient currents in LS neurons upon optogenetic stimulation of *Dat1*⁺ terminals expressing ChR2. **d,** Examples (LS) showing the lack of activation upon light stimulation of ChR2-expressing *Dat1*⁺ terminals (*n* = 13 out of 24 cells). **e,** Electrophysiological profiling of LS neurons responding to the light stimulation of ChR2-expressing *Dat1*⁺ terminals (*n* = 11 out of 24 cells). **f,** Representative voltage clamp-recordings demonstrating that blue light itself does not induce any inward transient currents in LS neurons in *Dat1*-Cre animals injected with AAV-DIO-mCherry in the PeVN instead of AAV-DIO-hChR2-mCherry (*n* = 11 cells from 3 mice). We used Biorender to visualize an experimental scheme in (**f**).

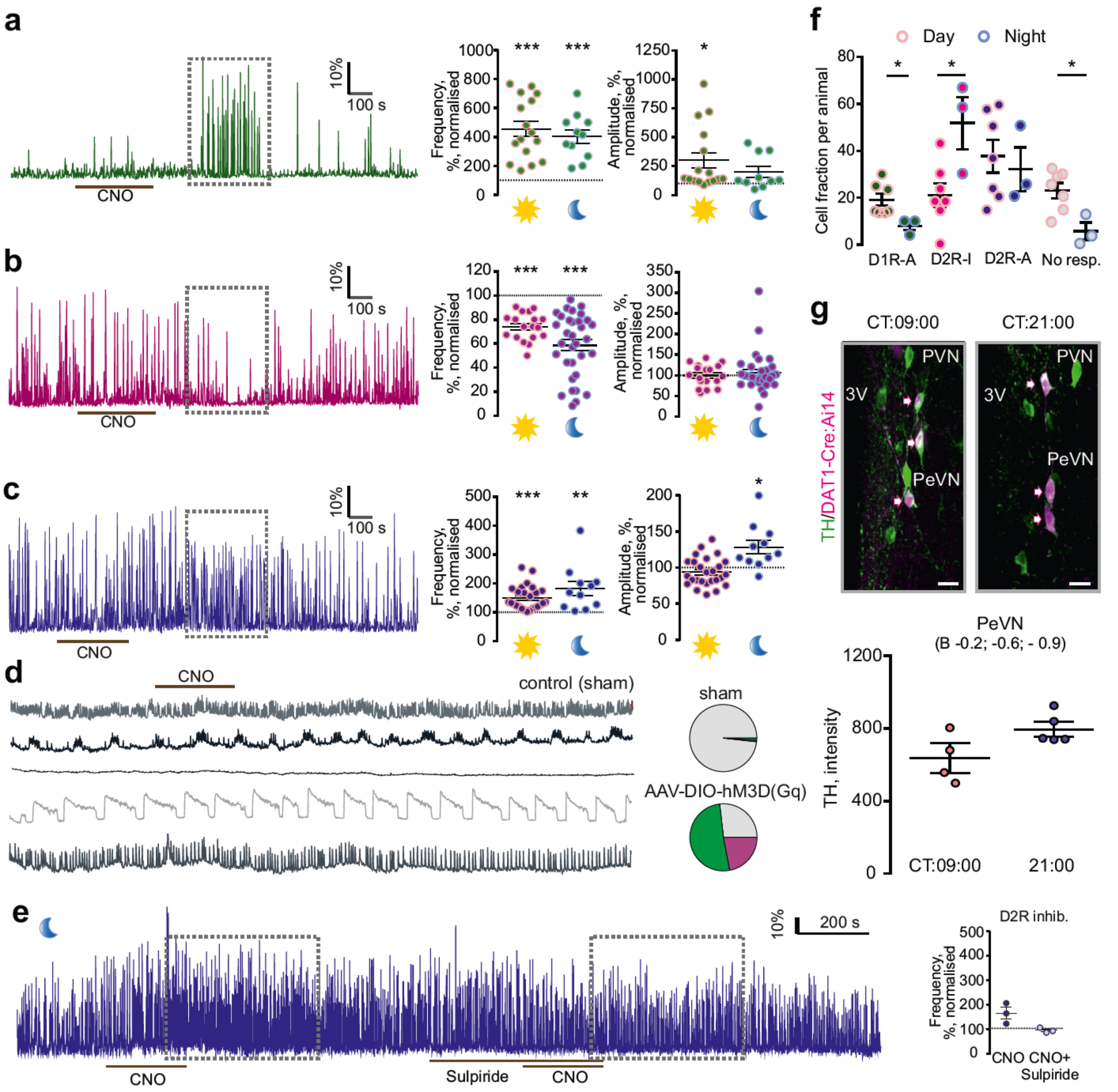

Supplementary Fig. 5. *Dat1*⁺ neurons modulate neuronal activity in the LS. **a-c,** CNO stimulation of *Dat1*⁺ terminals led to a change in the frequency and amplitude of Ca^{2+} oscillations in LS neurons. Colored traces (*left*) show representative examples of Ca^{2+} recordings at the single-cell level. *Right*: Data from individual recordings showing stimulatory/inhibitory effects. Day (sun symbol) and night (moon symbol) denote the timing of experiments. Data were normalized to controls (individual values) and presented as mean ± SEM. The statistical significance of the changes was analyzed with a one-sided paired t-test. * - p ≤ 0.05, ** - p ≤ 0.01, *** - p ≤ 0.001. n – the number of cells examined over at least three independent experiments (3 experiments for nights and 7 experiments for days). For (a) n = 16 for day, p = 0.000335 (frequency), p = 0.0019 (amplitude) and n = 11 cells for night, p = 0.00049 (frequency), p = 0.084 (amplitude). For (b) n = 18 for day, p = 1.83E-07 (frequency), p = 0.15215 (amplitude) and n = 33 cells for night, p = 2E-8 (frequency), p = 0.39687 (amplitude). For (c) n = 26 for day, p = 7.06E-09 (frequency), p = 0.03293 (amplitude) and n = 11 cells for night, p = 0.00334 (frequency), p = 0.04147 (amplitude). **d,** The effects of CNO on C^{a2+} signaling in LS neurons. Even though CNO was applied to the bath solution, it did not cause reversible changes in the activity of LS neurons in naïve *Dat1*-Cre animals (that is, without AAV-DIO-hM3D(Gq) injection into the PeVN). Grey color in the pie chart corresponds to unchanged activity, purple color denotes

reduced activity, while green color shows increased activity in *n* = 291 cells from *n* = 3 sham-injected animals. **e,** The analysis of sulpiride-induced D2R inhibition on the CNO-stimulated modulation of Ca^{2+} oscillations during the dark phase in the group of neurons characterized by D2R-dependent activation. n = 3 cells from 3 independent experiments. Data are presented as mean ± SEM. The statistical analysis was performed using two-sided paired t-test, p = 0.16517. **f,** Distribution of the four neuronal subtypes in the LS that produced differential responses upon the diurnal chemogenetic stimulation of *Dat1*$^{+}$ afferents. Individual data points were presented for each subject. Data are presented as mean ± SEM. The average data for each independent experiment (n =3 for night, n= 7 for day) are present as individual values. Two-sided unpaired t-test was used to estimate statistically significant changes in cell composition between day and night. * - p ≤ 0.05, ** - p ≤ 0.01. For D1-activated group p = 0.03; for D2-inhibited group p = 0.018; for D2-activated group p = 0.667, for non-responsive cells p = 0.014. **g,** TH levels in *Dat1*$^{+}$ neurons of the PeVN during day and night. Scale bar = 50 μm. *Abbreviations*: 3V, third ventricle; PVN, paraventricular nucleus. n = 4 and 5 animals for CT 09:00 and CT 21:00, respectively. The two-sided unpaired t-test showed no significant difference (p = 0.0523).

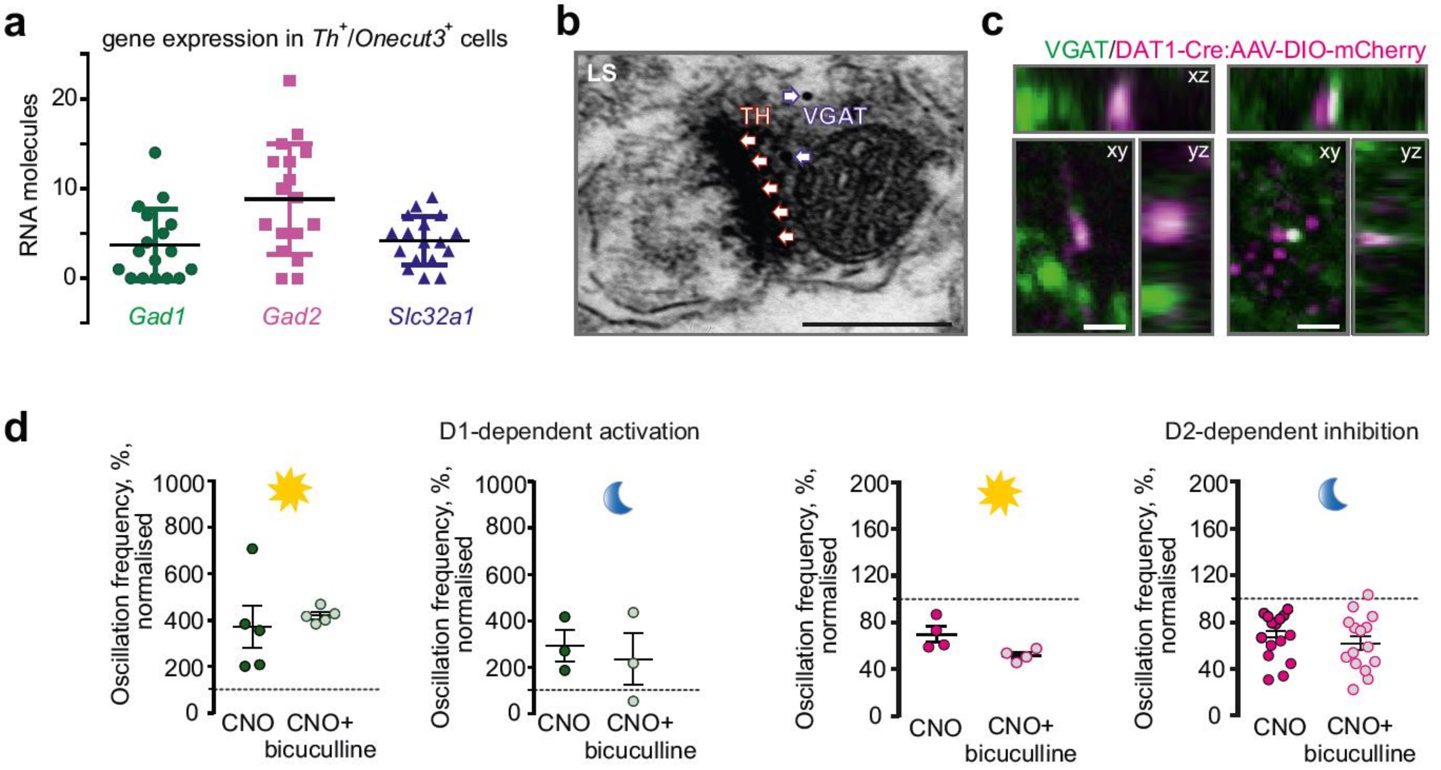

Supplementary Fig. 6. GABA neurotransmission does not contribute to the entrainment of the LS. a, Expression of genes related to GABA neurotransmission in *Th*⁺/*Onecut3*⁺ neurons. Data from juvenile animals (P10 and P23) were analyzed in an open-source single-cell RNA-seq dataset[40]. Data are presented as mean ± SD emerged with individual data points. n = 17 cells. **b,** Co-labeling for TH (DAB precipitate) and VGAT (immunogold) in the LS at the ultrastructural level. The postsynaptic density is marked by arrows. Scale bar = 0.5 μm. **c,** High-resolution orthogonal image stacks visualizing VGAT⁺ nerve terminals in the LS. The experiment was independently repeated 3 times with similar results. **d,** The contribution of $GABA_A$ receptors to responses of LS neurons (grouped in Fig. 4b-e) upon chemogenetic stimulation of *Dat1*⁺ terminals. Bicuculline at a concentration of 50 μM was used as a $GABA_A$ inhibitor. Sun symbol (light phase, CT: 06:00 – 11:00) and moon symbol (dark phase, CT: 18:00 – 23:00) correspond to the timing of the experiments. Error bars show mean ± SEM. n = 5 cells tested for the D1-dependently activated group during the light phase; n = 3 cells tested for the D1-dependently activated group during the dark phase. n = 4 cells tested for the D2-dependently inhibited group during the light phase. n = 15 cells tested for the D2-dependently inhibited group during the dark phase.

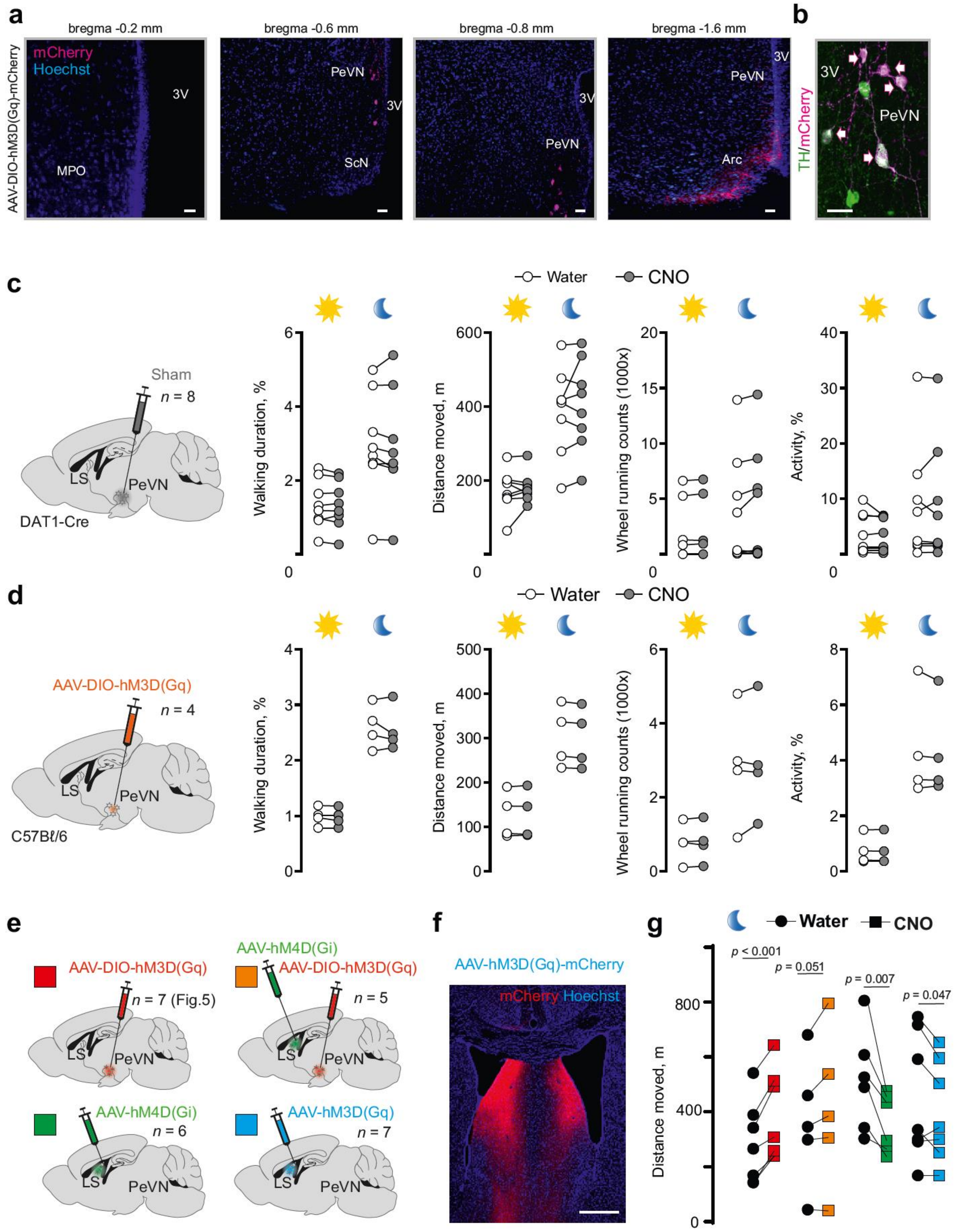
a
bregma -0.2 mm
bregma -0.6 mm
bregma -0.8 mm
bregma -1.6 mm
AAV-DIO-hM3D(Gq)-mCherry
mCherry
Hoechst
3V
MPO
PeVN
ScN
Arc
b
TH/mCherry
c
Water
CNO
Sham
n = 8
LS
PeVN
DAT1-Cre
Walking duration, %
Distance moved, m
Wheel running counts (1000x)
Activity, %
d
AAV-DIO-hM3D(Gq)
n = 4
C57Bℓ/6
e
AAV-DIO-hM3D(Gq)
n = 7 (Fig.5)
AAV-hM4D(Gi)
n = 5
n = 6
AAV-hM3D(Gq)
n = 7
f
AAV-hM3D(Gq)-mCherry
mCherry Hoechst
g
p < 0.001
p = 0.051
p = 0.007
p = 0.047
Distance moved, m

Supplementary Fig. 7. Contribution of *Dat1*[+] neurons in the anterior PeVN to the modulation of locomotion. a-b, *Post-hoc* analysis of *Dat1*-Ires-Cre mice used for the behavioral assessment (*see* Fig. 5a) and injected with AAV-DIO-hM3D(Gq)-mCherry in the PeVN. Representative images show cell distribution (**a**) and TH expression (**b**). The immunohistochemical analysis was done for each viral injection (7 independent experiments with similar results). Scale bars = 50 μm. **(c)** Control injections of vehicle into the PeVN of *Dat1*-Ires-Cre animals with subsequent behavioral analysis of locomotor activity (with/without CNO; *n* = 8 animals). The moon and sun symbols correspond to those in Fig. 5. **d,** Injection of AAV-DIO-hM3D(G_q) into the PeVN of wild-type animals and behavioral assessment of the effects of CNO administration on locomotion (*n* = 4 animals). **e,** Experimental design of *in vivo* behavioral experiments to identify the downstream role of the LS in PeVN-driven changes of locomotor activity. **f,** Representative image shows the spread of Cre-independent virus particles in the LS. The immunohistochemical analysis was done for each viral injection (7 independent experiments with similar results for AAV-hM3D(Gq)). Scale bar = 1 mm. **g,** The effect of the CNO on locomotor activity (distance moved) during the dark phase (CT: 12:00-18:00). Colors and n-s are equivalent to those shown in (**e**). A one-sided paired t-test was used to assess statistically significant changes upon CNO administration. p = 0.000158 for chemogenetic activation of DAT+ A14 cells (red), p=0.0509 for the simultaneous chemogenetic activation of A14 cells and inhibition of LS neurons (orange); p = 0.00739 in case of chemogenetic inhibition of LS neurons (green); p = 0.0473 for chemogenetic activation of LS neurons (blue). We used Biorender to visualize experimental schemes in (**c,d,e**).

Supplementary Table 1. List of AAV viruses used in this study

ID	Name	Supplier	Activity	Category
**50459-AAV8**	pAAV-hSyn-DIO-mCherry	Addgene, a plasmid was provided by Bryan Roth	mCherry, Cre-dependent	Tracer
**100043-AAV9**	pAAV-hSyn-DIO-EGFP.WPRE.hGH	Addgene, a plasmid was provided by Ian Wickersham	EGFP, Cre-dependent	Tracer
**59462-AAVrg**	pAAV-CAG-tdTomato	Addgene, a plasmid was provided by Edward Boyden	Retrograde tdTomato	Tracer
**20297-AAV8**	pAAV-EF1a-double floxed-hChR2(H134R)-mCherry-WPRE-HGHpA	Addgene, a plasmid was provided by Karl Deisseroth	Activator, Cre-dependent	Optogenetics
**51085-AAV1**	AAV-hSyn1-GCaMP6f-P2A-nls-dTomato	Addgene, a plasmid was provided by Jonathan Ting	GCaMP6f and physically separate nuclear dTomato	Calcium sensor
**44361-AAV8**	pAAV-hSyn-DIO-hM3D(Gq)-mCherry	Addgene, a plasmid was provided by Bryan Roth	Activator, Cre-dependent	DREADD
**44362-AAV8**	pAAV-hSyn-DIO-hM4D(Gi)-mCherry	Addgene, a plasmid was provided by Bryan Roth	Inhibitor, Cre-dependent	DREADD
**67845**	pAAV-hsyn-flex-dsRed-shvgat	Addgene, a plasmid was provided by William Wisden	shRNA, Cre-dependent	Gene silencing

Supplementary Table 2. List of antibodies

Antibody	Source	Concentration	Supplier& Cat. №
**Polyclonal-mCherry**	chicken	1:1,000	EnCor Biotech, Cat. № CPCA-mCHERRY
**ONECUT3**	guinea pig	1:3,000	Dr. G. G. Rousseau
**Tyrosine hydroxylase**	rabbit	1:300	Millipore, Cat. № AB152
**Tyrosine hydroxylase**	mouse	1:500	Millipore Cat. № MAB5280, clone 2/40/15
**Phospho-Ser^{40}-TH**	rabbit	1:1,000	Millipore, Cat. № AB5935
**Neuromedin S**	rabbit	1:1,000	Bachem, Cat. № T-4814.0400
**GFP**	goat	1:1,000	Abcam, Cat. № AB6662
**VGAT**	guinea pig	1:500	Synaptic Systems, Cat. № 131 004
**VGAT**	rabbit	1:100	Synaptic Systems, Cat. № 131 003
**VMAT2**	rabbit	1:1,000	Synaptic Systems, Cat. № 138 302
**Somatostatin**	rat	1:250	Millipore, Cat. № MAB354 , clone YC7
**Hoechst 33,342**		1:10,000	Sigma, Cat. № B2261

Supplementary Table 3. Imaging specifications for light-sheet microscopy

Figure	Animal line	System	Objective	Image pixel size	z-step	Imaging depth	Acquisition direction
**Fig. 2b**	DAT-Ires-Cre	UM	corr. 4xN.A. 0.28 RI: 1.56	2.59 µm x 2.59 µm	2.59 µm	2807 µm	from sagittal
**Fig. 2c**	DAT-Ires-Cre	UM	Leica 16x N.A. 0.6 RI:1.56	0.6 µm x 0.6 µm	1.2µm	1563 µm	from ventral
**Suppl.Fig. 3a**	DAT-Ires-Cre	UM	Leica 16x N.A. 0.6 RI:1.56	0.6 µm x 0.6 µm	1.2µm	1502 µm	from ventral

UM: ultramicroscope= light sheet microscope; corr.: corrected
