# Supplementary material for: A hypothalamic dopamine locus for psychostimulant-induced hyperlocomotion in mice
Source: Nat Commun. 2022 Oct 8;13:5944. doi: 10.1038/s41467-022-33584-3 (PMC9547883; doi:10.1038/s41467-022-33584-3)
Supplement: Supplementary file 2 — Description of Additional Supplementary Files [file 41467_2022_33584_MOESM2_ESM.pdf]

Description of Additional Supplementary Files

File Name: Supplementary Movie 1

Description: 3D reconstruction of neuronal projections of A14 neurons in the lateral septum.

File name: Supplementary Movie 2

Description: 3D reconstruction of neuronal projections of A14 neurons in the central amygdala.
