# Supplementary material for: A hypothalamic dopamine locus for psychostimulant-induced hyperlocomotion in mice
Source: Nat Commun. 2022 Oct 8;13:5944. doi: 10.1038/s41467-022-33584-3 (PMC9547883; doi:10.1038/s41467-022-33584-3)
Supplement: Supplementary file 5 — Reporting Summary [file 41467_2022_33584_MOESM5_ESM.pdf]

nature research

Corresponding author(s): Tibor Harkany, Roman Romanov

Last updated by author(s): Aug 26, 2022

Reporting Summary

Nature Research wishes to improve the reproducibility of the work that we publish. This form provides structure for consistency and transparency in reporting. For further information on Nature Research policies, see our Editorial Policies and the Editorial Policy Checklist.

Statistics

For all statistical analyses, confirm that the following items are present in the figure legend, table legend, main text, or Methods section.

n/a	Confirmed	
☐	☒	The exact sample size (*n*) for each experimental group/condition, given as a discrete number and unit of measurement
☐	☒	A statement on whether measurements were taken from distinct samples or whether the same sample was measured repeatedly
☐	☒	The statistical test(s) used AND whether they are one- or two-sided<br>*Only common tests should be described solely by name; describe more complex techniques in the Methods section.*
☒	☐	A description of all covariates tested
☐	☒	A description of any assumptions or corrections, such as tests of normality and adjustment for multiple comparisons
☐	☒	A full description of the statistical parameters including central tendency (e.g. means) or other basic estimates (e.g. regression coefficient) AND variation (e.g. standard deviation) or associated estimates of uncertainty (e.g. confidence intervals)
☐	☒	For null hypothesis testing, the test statistic (e.g. *F*, *t*, *r*) with confidence intervals, effect sizes, degrees of freedom and *P* value noted<br>*Give P values as exact values whenever suitable.*
☒	☐	For Bayesian analysis, information on the choice of priors and Markov chain Monte Carlo settings
☒	☐	For hierarchical and complex designs, identification of the appropriate level for tests and full reporting of outcomes
☒	☐	Estimates of effect sizes (e.g. Cohen's *d*, Pearson's *r*), indicating how they were calculated

Our web collection on statistics for biologists contains articles on many of the points above.

Software and code

Policy information about availability of computer code

Data collection	In vitro electrophysiology: data were collected with PatchMaster 2.80.<br>In vitro calcium measurements were collected using VisiView 3.0.3.0 software (Visitron Systems).<br>Behavioural data were collected using EthoVision XT15 (Noldus IT).<br>Confocal images were taken using the ZEN2013 software (Zeiss).<br>Image processing from light-sheet microscopy was done using MATLAB 9 (MathWorks, USA) with implemented contrast-limited adaptive histogram equalisation and Fast-Fourier-Transform-based destriping algorithm and Amira 6.7 software (Thermofisher, USA) for 3D image reconstruction (ref.91: Hahn et al 2019).<br>Photoshop CS6 (Adobe) was used to crop the original images. Multi-panel figures were assembled in CorelDraw X9 (Corel Corp.).
Data analysis	In vitro electrophysiology and calcium imaging data were analyzed in Clampfit 10.0 (Molecular Devices).<br>Confocal images were analyzed with the ZEN2013 software.<br>Behavioural data were scored in Ethovision XT15.<br>Statistical tests were performed with GraphPad Prism 7 and SigmaPlot13.0.<br>Data from single-cell RNA-sequencing were re-processed and re-analyzed from open-label sources using the Seurat V3 and tidyverse packages in R.

For manuscripts utilizing custom algorithms or software that are central to the research but not yet described in published literature, software must be made available to editors and reviewers. We strongly encourage code deposition in a community repository (e.g. GitHub). See the Nature Research guidelines for submitting code & software for further information.

Data

Policy information about availability of data

All manuscripts must include a data availability statement. This statement should provide the following information, where applicable:

- Accession codes, unique identifiers, or web links for publicly available datasets
- A list of figures that have associated raw data
- A description of any restrictions on data availability

Single-cell RNA-seq data were published earlier and can be downloaded in raw and processed forms from the NCBI Gene Expression Omnibus database. Accession numbers are GSE74672 (https://www.ncbi.nlm.nih.gov/geo/query/acc.cgi?acc=GSE74672), GSE132730 (https://www.ncbi.nlm.nih.gov/geo/query/acc.cgi?acc=GSE132730). Data generated in this study are available in the Source Data file.

Field-specific reporting

Please select the one below that is the best fit for your research. If you are not sure, read the appropriate sections before making your selection.

[x] Life sciences [ ] Behavioural & social sciences [ ] Ecological, evolutionary & environmental sciences

For a reference copy of the document with all sections, see nature.com/documents/nr-reporting-summary-flat.pdf

Life sciences study design

All studies must disclose on these points even when the disclosure is negative.

Sample size	The sample size was chosen according to accepted practices in the field of Neuroscience. We estimated the necessary sample size based on preliminary experiments and previous studies using similar animal models (Romanov et al, 2017; Romanov et al, 2020; Stagkourakis S et al, 2018; Stagkourakis S et al, 2020; Zhang X, van den Pol AN., 2016) which provided clear suggestions for the number of animals sufficient to power the statistical tests per each experiment. For our research on animals, we have made every effort to avoid unnecessary animal use and to reach statistical significance with minimally necessary group sizes when using one-sided unpaired/paired t-test, Mann-Whitney test (in case normality test failed) or one-way ANOVA for multiple groups. Therefore, and considering the typical variance in mouse behavior, to assure reproducibility we relied on re-sampling, parallel processing and repetitive iterations to reach statistical power when applying the appropriate statistical test with n = 5-8 animals per group. Histological quantitative analyses were performed using a minimum group size of n = 3/ variable/time-point. Electrophysiological and calcium imaging data were collected from a minimum of n = 10 mice (n = 3 mice for additional sham controls to test pure effects of CNO and blue light on the electrophysiological and calcium activities).
Data exclusions	Animals injected with AAV viruses that showed no detectable virus-mediated gene expression in the target region and/or presented ectopic labeling upon immunohistochemical analysis or post-hoc immune-identification were excluded. DAT positive neurons situated in the anterior tip of the periventricular area (bregma: 0.3 – 0.2 mm) were excluded from our analysis due to ambiguities in accurately discriminating A14 periventricular neurons from A15 dopamine cells based on TH/DAT expression alone. In whole-cell patch clamp recordings, access resistance was monitored throughout the experiments, and neurons in which the series resistance exceeded 15 MΩ or changed ≥25% were excluded.
Replication	Behavioural experiments were performed in batches of 2 or 4 animals and invariably included as many animals with a given experimental manipulation per batch (parallel processing) and among individuals that underwent repetitive iterations. Electrophysiological and calcium measurements were replicated at least three times in independent experiments. Anatomy findings were replicated in two and more independent experiments.
Randomization	Animals were randomly allocated to the experimental and control groups in all experiments. Mice from the same litter were assigned to different test groups. Mice were randomly settled in PhenoTyper cages. For amphetamine treatment, the sequence of individual chemogenetic challenges and amphetamine injections alternated between the batches of mice used.
Blinding	These experiments cannot be considered blinded because the same individuals were involved in all stages of the specific experiments (i.e. researchers who performed the experiments also analyzed the ensuing dataset(s)).

Reporting for specific materials, systems and methods

We require information from authors about some types of materials, experimental systems and methods used in many studies. Here, indicate whether each material, system or method listed is relevant to your study. If you are not sure if a list item applies to your research, read the appropriate section before selecting a response.

Materials & experimental systems

n/a	Involved in the study	
	x	Antibodies
x		Eukaryotic cell lines
x		Palaeontology and archaeology
	x	Animals and other organisms
x		Human research participants
x		Clinical data
x		Dual use research of concern

Methods

n/a	Involved in the study	
x		ChIP-seq
x		Flow cytometry
x		MRI-based neuroimaging

Antibodies

Antibodies used

Primary:
rabbit anti-TH (1:300; Millipore Cat. № AB152);
rabbit anti-phospho-Ser40-TH (1:1,000; Millipore, Cat. № AB5935);
guinea pig anti-ONECUT3 (1:3,000; donated by Dr. G. G. Rousseau);
goat anti-GFP (1:1,000; Abcam Cat. № ab6662);
chicken anti-mCherry (1:1,000; EnCor Biotechnology Cat. № CPCA-mCherry);
rat anti-somatostatin (1:250; Millipore, Cat. № MAB354, clone YC7);
rabbit anti-neuromedin S (1:1,000; Bachem, Cat. № T-4814.0400);
guinea pig anti-VGAT (1:500; Synaptic Systems, Cat. № 131 004);
rabbit anti-VMAT2 (1:1,000; Synaptic Systems, Cat. № 138 302);
mouse anti-TH (1:500; Millipore Cat. № MAB5280, clone 2/40/15);
rabbit anti-VGAT (1:100; Synaptic Systems, Cat. № 131 003/28).

Secondary:
Cy3-AffiniPure Donkey Anti-Chicken (1:300; Jackson ImmunoResearch Labs, Cat. № 703-165-155);
Cy2-AffiniPure Donkey Anti-Rabbit (1:300;Jackson ImmunoResearch Labs Cat. № 711-225-152);
Alexa Fluor 488-AffiniPure Donkey Anti-Goat (1:300;Jackson ImmunoResearch Labs Cat. № 705-545-147);
Alexa Fluor 647-AffiniPure Donkey Anti-Guinea Pig (1:300;Jackson ImmunoResearch Labs Cat. № 706-605-148);
Alexa Fluor Cy-5 Donkey Anti-Guinea Pig (1:300;Jackson ImmunoResearch Labs Cat. № 706-175-148)
Alexa Fluor 647-AffiniPure Donkey Anti-Rabbit (1:300;Jackson ImmunoResearch Labs Cat. № 711-605-152);
Alexa Fluor 647-AffiniPure Donkey Anti-Rat (1:300;Jackson ImmunoResearch Labs Cat. № 712-605-153);
Biotin-SP (long spacer) AffiniPure Donkey Anti-Mouse IgG (H+L) (1:1000; Jackson ImmunoResearch Labs Cat. № 715-065-151);
EM Goat anti-Rabbit IgG: 15nm Gold (1:15; BBI Solutions, Cat. № EM.GAR15, Patch 8023).

Validation

For goat anti-GFP and chicken anti-mCherry:
Species: Mouse; Applications: IHC;
Kastriti, M. E. et al. Schwann cell precursors generate the majority of chromaffin cells in zuckerkandl organ and some sympathetic neurons in paraganglia. Front. Mol. Neurosci. 12, 6 (2019).

For guinea pig anti-ONECUT3:
Species: Mouse; Applications: IHC;
Espana, A. & Clotman, F. Onecut transcription factors are required for the second phase of development of the A13 dopaminergic nucleus in the mouse. J. Comp. Neurol. 520, 1424–1441 (2012).

For rabbit anti-VMAT2:
Species: Mouse; Applications: IHC;
Fortune, T., Lurie, D.I. Chronic low-level lead exposure affects the monoaminergic system in the mouse superior olivary complex. The Journal of comparative neurology. 5135, 542-58 (2009).

For guinea pig anti-VGAT:
Species: Mouse; Applications: IHC;
Santos, M., D'Amico, D., Spadoni, O., Amador-Arjona, A., Stork, O., Dierssen, M. Hippocampal hyperexcitability underlies enhanced fear memories in TgNTRK3, a panic disorder mouse model. The Journal of neuroscience. 3338, 15259-71 (2013).

For rabbit anti-VGAT:
WB, IHC; tested species: mouse
Seigneur E, Südhof TC. Genetic Ablation of All Cerebellins Reveals Synapse Organizer Functions in Multiple Regions Throughout the Brain. The Journal of neuroscience. 3820: 4774-4790 (2018).

For rabbit anti-TH, rabbit anti-phospho-Ser40-TH and rabbit anti-neuromedin S:
Species: Mouse; Applications: IHC;
Romanov, R. A. et al. Molecular interrogation of hypothalamic organization reveals distinct dopamine neuronal subtypes. Nat. Neurosci. 20, 176–188 (2017).

For mouse anti-TH antibody:
Species: Mouse, Application: IHC;
Tatti, R; Bhaukaurally, K; Gschwend, O; Seal, RP; Edwards, RH; Rodriguez, I; Carleton, A. A population of glomerular glutamatergic neurons controls sensory information transfer in the mouse olfactory bulb. Nature communications 5 , 3791 (2014).
Wang, L; Mogami, S; Yakabi, S; Karasawa, H; Yamada, C; Yakabi, K; Hattori, T; Taché, Y. Patterns of Brain Activation and Meal Reduction Induced by Abdominal Surgery in Mice and Modulation by Rikkunshito. PloSOne 10, e0139325 (2015).

For rat anti-Somatostatin:
Species: Mouse; Applications: IHC;
Liu, X., Dimidschstein, J., Fishell, G., Carter, A. G. Hippocampal inputs engage CCK+ interneurons to mediate endocannabinoid-modulated feed-forward inhibition in the prefrontal cortex. Elife. e55267 (2020).

Animals and other organisms

Policy information about studies involving animals; ARRIVE guidelines recommended for reporting animal research

Laboratory animals	DAT-Ires-Cre (IMSR Cat. № JAX:006660);<br>Ai14 (IMSR Cat. № JAX:0079);<br>Mus musculus C57BL/6J (IMSR Cat. № JAX:000664);<br>Tg(Th-EGFP)21-31Koba (Riken Bioresource Center);<br>Drd1-EGFP and Drd2-EGFP (GENSAT, The Rockefeller University).<br>Young adult males and females at the age of P21-P40 were used in this study.
Wild animals	No wild animals were used in the study.
Field-collected samples	No field-collected samples were used in the present study.
Ethics oversight	All procedures involving laboratory animal, such as experiments, caretaking and housing/husbandry were in strict compliance with the ethical regulations of the 2010/63/EU European Communities Council Directive and approved by the Austrian Ministry of Science and Research (66.009/0145-WF/II/3b/2014, and 66.009/0277-WF/V/3b/2017). Regulations on pre- and post-operative care, particularly the use of analgesics and anaesthetics to minimize any discomfort and pain to animals, were also followed. The 3Rs rule (Replacement, Reduction and Refinement) was systematically applied throughout this study (both design and execution).

Note that full information on the approval of the study protocol must also be provided in the manuscript.
